# Molecular Evolution of the Chikungunya Virus *E1* Gene in Saudi Arabia: Predominance of Purifying Selection and ECSA/IOL Lineage Circulation

**DOI:** 10.3390/v18070791

**Published:** 2026-07-19

**Authors:** Mohamed A. Farrag

**Affiliations:** Department of Botany and Microbiology, College of Science, King Saud University, Riyadh 11451, Saudi Arabia; mfarrag@ksu.edu.sa

**Keywords:** Chikungunya virus, *E1* gene, selection pressure, purifying selection, molecular epidemiology, ECSA genotype

## Abstract

Background: Chikungunya virus (CHIKV) is a re-emerging alphavirus that has caused millions of cases worldwide, yet its molecular epidemiology in Saudi Arabia remains poorly understood. This study integrates bioinformatic analysis of the envelope gene (*E1*) gene sequences from Saudi isolates with global genotypes to characterize circulating lineages, selection pressures and stability effects of endemic mutations. Methods: A total of 109 CHIKV *E1* sequences (1155 bp) representing the East/Central/South African (ECSA), Asian, and West African genotypes were retrieved from GenBank and GISAID. Phylogenetic relationships were reconstructed using maximum likelihood (IQ-TREE). Codon-based selection analyses were performed with MEME, FEL, SLAC, and FUBAR. The structural effects of nine missense mutations were assessed using DynaMut and consensus predictors (DUET, mCSM). Results: All seven Saudi isolates clustered within the ECSA-Indian Ocean Lineage (IOL) subclade with strong bootstrap support (≥95%). Short branch lengths among Saudi strains indicated recent common ancestry and limited local divergence, suggesting possible repeated introductions or limited local circulation. No codon showed robust evidence of positive selection across multiple methods. However, episodic diversifying selection was detected at codon 99 (MEME, *p* = 0.01), while pervasive purifying selection acted on numerous sites (e.g., codons 135, 307, 344; strong signals across FEL, SLAC, and FUBAR). A single conserved N-linked glycosylation site was present at residue 141 (NITV motif) in all Saudi and most global strains. Three mutations unique to or prominent in Saudi isolates (N20H, L136F, A249T) were identified; consensus stability predictions (DUET) classified them as destabilizing. Conclusions: Saudi CHIKV strains belong exclusively to the ECSA-IOL lineage and exhibit strong purifying selection on the *E1* gene, consistent with functional constraints on this essential fusion protein. The identified Saudi-associated mutations appear to be non-adaptive, tolerated changes. These findings underscore the value of continued genomic surveillance to monitor potential adaptive evolution, particularly in the context of mass gatherings and competent *Aedes* vectors.

## 1. Introduction

CHIKV is an arthropod-borne alphavirus belonging to the family Togaviridae. Its positive-sense single-stranded RNA genome is approximately 11.8 kb in length and encodes four non-structural proteins (*nsP1*–*nsP4*) and five structural proteins (*capsid*, *E3*, *E2*, *6K* and *E1*) [1,2,3]. The envelope glycoproteins *E2* and *E1* are critical for virus entry. *E2* mediates attachment to host cell receptors, while *E1* is a class II fusion protein that drives fusion of the viral and endosomal membranes, allowing release of the nucleocapsid into the cytoplasm [4,5]. The mature virion displays 80 trimeric spikes on its surface, each spike composed of three *E1–E2* heterodimers [6,7]. The *E1* protein is a 415-amino-acid polypeptide folded into three β-sheet-rich domains (I, II and III), with a highly conserved fusion loop in domain II that is essential for the low-pH-induced conformational change required for membrane fusion [5,8].

The virus circulates in three major genotypes: the West African, the ECSA and the Asian genotype [9,10]. A fourth lineage, the Indian Ocean lineage (IOL), is now recognized as a distinct sub-lineage of the ECSA genotype [11,12]. CHIKV originated in Africa and has spread globally over the past six decades, causing explosive outbreaks that have infected millions of people [13,14,15]. The global expansion has been particularly dramatic in the 21st century: the IOL has spread from coastal Kenya to Indian Ocean islands, the Indian subcontinent, Southeast Asia and subsequently to Europe and the Americas, with the Americas alone reporting over 13 million suspected cases in 2024 [16,17]. By late 2025, over 500,000 confirmed and suspected cases were recorded worldwide [18,19]. CHIKV now poses a threat to nearly 2.8 billion people across more than 100 countries [20]. The recent large-scale outbreaks have been driven in large part by the acquisition of the *E1*-A226V substitution in the IOL, which dramatically increases the virus’s infectivity for Aedes albopictus mosquitoes [21,22,23].

The molecular epidemiology of CHIKV in Saudi Arabia has only recently begun to be explored. The first autochthonous case of CHIKV in the Kingdom was reported in May 2011 in Jeddah, a 55-year-old woman presenting with severe arthralgia, fever, rash and myalgia, with no history of travel, confirming local transmission [24]. Subsequent genomic characterization of isolates collected in Jeddah in 2018 revealed that these strains belong to the ECSA-IOL subclade and show high genetic similarity to strains circulating in Mombasa, Kenya, in 2017–2018, strongly suggesting multiple introductions of the virus from East Africa [25]. A more recent case in 2021 in Jeddah, involving a 32-year-old male of Indian origin, gave rise to a genetically distinct ECSA-IOL strain belonging to the Indian subcontinent/Southeast Asia clade [26]. Key mutations identified in these Saudi isolates include *E1*-L136F, *E1*-K211E and *E1*-I317V, together with several novel substitutions [26]. Despite the presence of competent Aedes vectors in the western coastal region and the enormous influx of pilgrims (over two million each year during Hajj, arriving from more than 180 countries, many of which are CHIKV-endemic) [27], sustained transmission of CHIKV in Saudi Arabia appears to be limited. Only a handful of cases have been confirmed over the past decade, and seroprevalence studies suggest a low but measurable background seropositivity rate [28]. The reasons for this relative quiescence remain incompletely understood.

The *E1* protein is an attractive target for molecular epidemiological surveillance because it carries many of the adaptive mutations that govern vector specificity (e.g., A226V) and is subject to host-immune pressures [29,30]. However, comprehensive analyses of the selection pressures acting on the *E1* gene of CHIKV circulating in Saudi Arabia, together with systematic investigation of the effects of endemic mutations on protein stability, are currently lacking. In the present study, a thorough in silico characterization of all available *E1* gene sequences from Saudi isolates, together with representative global sequences from the three major genotypes, was performed. Specifically, the objectives of this study were: (i) to reconstruct the phylogenetic relationships of Saudi isolates relative to global reference strains; (ii) to perform codon-based selection pressure analyses using four complementary methods (MEME, FEL, SLAC and FUBAR); and (iii) to predict the effects of non-synonymous mutations on *E1* protein stability and flexibility using a panel of structure-based and sequence-based tools.

## 2. Materials and Methods

### 2.1. Study Design and Data Sources

**Figure 1:**
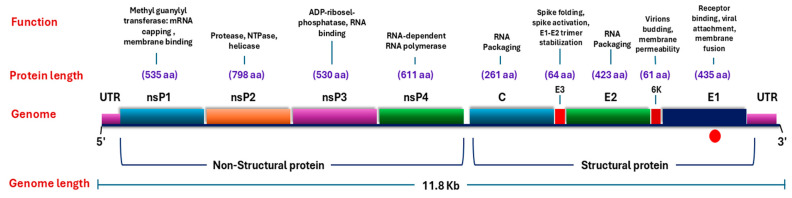
Schematic representation of the CHIKV genome organization. The positive-sense single-stranded RNA genome (~11.8 kb) encodes two open reading frames. The 5′ two-thirds encode the non-structural polyprotein (*nsP1*–*nsP4*), which is processed into proteins involved in viral replication and RNA capping. The 3′ one-third encodes the structural polyprotein, which is cleaved into capsid (C), *E3*, *E2*, *6K* and the *E1* envelope glycoprotein. The red circle highlights the *E1* gene, the target of the present study. *E1* is a class II fusion protein essential for viral entry and a key target for phylogenetic and selection-pressure analyses.

This in silico study performed a bioinformatics analysis of publicly available CHIKV *E1* gene (Figure 1) sequences from human cases in the Kingdom of Saudi Arabia, together with reference sequences from global genotypes.

### 2.2. Sequence Data Retrieval and Processing

A total of 109 CHIKV *E1* gene sequences (a 1155 nucleotides fragment of the *E1* gene) representing the three genotypes, ECSA (*n* = 73) (including 7 Saudi strains), Asian (*n* = 25) and West African (*n* = 11) genotypes, were retrieved from the National Center for Biotechnology Information (NCBI) GenBank and the Global Initiative on Sharing All Influenza Data (GISAID) platform. Reference prototype strain (AF369024-S27-1952) was also downloaded for comparative purposes. Sequence datasets were curated by excluding duplicate entries, short fragments, and sequences with high proportions of ambiguous bases. Specifically, the following filtering criteria were applied: (i) duplicate entries were defined as sequences with 100% nucleotide identity and identical metadata (country and year); (ii) short fragments were defined as sequences shorter than the target 1155 bp analysis region; and (iii) sequences with ≥1% ambiguous bases (Ns) were excluded. All sequences were manually inspected and adjusted as needed. Retrieved sequences were edited and assembled using BioEdit software (version 7.0). Multiple sequence alignments for the *E1* gene were initially performed via the Clustal W algorithm implemented in the MegAlign program (DNASTAR Lasergene). The alignments were manually inspected and adjusted as needed. All sequences used in this study are publicly available in GenBank/GISAID; accession numbers are listed in the Supplementary Materials (Table S1). The final dataset of 109 high-quality sequences was used for all downstream analyses.

### 2.3. Nucleotide and Amino Acid Sequence Analysis

The *E1* sequences were analyzed to determine pairwise percent identities and genetic divergence. Saudi sequences were compared against genotype-specific international reference strains retrieved from GenBank, using the respective prototype strains as consensus sequences. Nucleotide mutations and amino acid substitutions were identified and categorized as permanent (shared across all time periods), time-specific, genotype-specific, or potentially Saudi-unique. To predict potential glycosylation sites in the *E1* protein, N-glycosylation motifs (Asn-X-Ser/Thr) were identified using the NetNGlyc 1.0 server (https://services.healthtech.dtu.dk/services/NetNGlyc-1.0/, accessed on 20 April 2026), and O-glycosylation sites were predicted via the NetOGlyc 4.0 server (https://services.healthtech.dtu.dk/services/NetOGlyc-4.0/, accessed on 25 April 2026), both with default thresholds (0.5 for N-glycan and 0.5 for O-glycan predictions). Only residues with prediction scores above the respective thresholds were considered positive. Conserved N-glycosylation sites were defined as those present in all analyzed sequences, while O-glycosylation patterns were compared across isolates from different years to identify temporal changes.

### 2.4. Phylogenetic Analysis and Genotype Assignment

Phylogenetic reconstruction was performed on the final dataset of 109 CHIKV *E1* sequences (1155 bp alignment). Sequence datasets were curated by removing duplicates, short fragments, and sequences with high proportions of ambiguous bases. Multiple sequence alignment was performed using Clustal W implemented in MEGA11 (V10.2.6) [31] and manually adjusted using AliView (V1.31.1) [32]. The alignment was trimmed to remove the first 150 bp and last 45 bp, corresponding to the hypervariable signal peptide and transmembrane domains, resulting in a final alignment of 960 bp for phylogenetic analysis. The best-fit nucleotide substitution model was determined using the Bayesian Information Criterion (BIC) in MEGA11, which selected the General Time Reversible with Invariant sites (GTR+I) model (BIC = 13,695.527; lnL = −5532.526). Maximum likelihood phylogenetic trees were reconstructed using IQ-TREE version 3.1.3 [33] with 1000 bootstrap replicates under the GTR+I model. The final maximum likelihood tree was visualized and annotated using FigTree version 1.4.4 (http://tree.bio.ed.ac.uk/software/figtree/, accessed on 9 July 2026).

### 2.5. Assessment of Root-to-Tip Regression and Temporal Signal

To determine whether the *E1* gene dataset contained sufficient temporal structure for molecular clock calibration, a root-to-tip regression analysis was performed using TempEst v1.5.3. Pairwise genetic distances (calculated under the GTR+I model) were plotted against sampling dates (1953–2026) for all 109 sequences. The regression revealed a very weak negative correlation between genetic divergence and sampling time (R^2^ = 1.26 × 10^−3^; correlation coefficient = −0.0355), indicating a complete absence of usable temporal signal for reliable divergence time estimation.

### 2.6. Selection-Pressure Analysis

*E1* nucleotide sequences were aligned at the codon level using the MUSCLE algorithm [34] implemented in MEGA11 [35] and subsequently inspected and edited in AliView. To obtain an in-frame codon alignment, the protein alignment was generated and back-translated to the corresponding codon alignment using the kc-align tool (v.1.0.2+galaxy1) within the Galaxy platform [36] (https://usegalaxy.org, accessed on 10 May 2026). kc-align is a codon-aware multiple aligner that uses Kalign3 to produce in-frame gapped codon alignments suitable for selection analysis. The resulting codon alignment, comprising the 7 Saudi strains plus the three-genotype background sequences, was subjected to positive-selection analysis using the Datamonkey 2.0 web server [37] (https://www.datamonkey.org/, accessed on 10 May 2026), which implements the HyPhy package [38]. Four complementary methods were employed: the Mixed Effects Model of Evolution (MEME) [39] to detect episodic diversifying selection; the Fixed Effects Likelihood (FEL) [38] and the Single-Likelihood Ancestor Counting (SLAC) (Kosakovsky Pond & Frost, 2005) to detect pervasive selection; and the Fast Unconstrained Bayesian AppRoximation (FUBAR) [39] to provide a Bayesian estimate of pervasive selection. A site was considered positively selected if the *p*-value was <0.1 in MEME, FEL and SLAC, or the posterior probability was >0.9 in FUBAR, and the site was identified by at least two of the four methods.

### 2.7. Protein Stability Prediction

The potential impact of missense mutations identified in the CHIKV *E1* protein on structural stability was assessed using the DynaMut web server (http://biosig.unimelb.edu.au/dynamut/, accessed on 15 May 2026), which combines normal mode analysis (NMA) with graph-based signatures to predict stability changes upon mutation [40]. Since no high-resolution crystal structure was available for the specific Saudi strain variants, a three-dimensional structural model of the wild-type *E1* protein was first generated via comparative (homology) modeling using the SWISS-MODEL server (https://swissmodel.expasy.org/, accessed on 16 May 2026) based on the prototype ECSA strain S27 sequence (GenBank accession AF485728). Model quality was assessed using the Global Model Quality Estimation (GMQE = 0.88) and QMEANDisCo Global score (0.83 ± 0.05), with stereochemical quality validated by Ramachandran plot analysis. The resulting model was used as the reference structure. For each of the nine missense substitutions identified in the Saudi CHIKV strains (N20H, L136F, A249T, K211E, K211N, M269V, D284E, I317V, and V322A), the specific mutation was introduced in silico into the reference PDB model, and the mutated structure was submitted to DynaMut. The server calculated the primary DynaMut stability score (ΔΔG, kcal/mol), the normal mode analysis-based ENCoM score (ΔΔG ENCoM, kcal/mol), and the structure-based predictions from mCSM, SDM and the consensus DUET method. Positive ΔΔG values were interpreted as stabilizing, negative values as destabilizing. All mutations were located in loop or irregular secondary structure regions according to the structural annotation. The predicted stability changes for the three Saudi-unique mutations (N20H, L136F, A249T) were compiled for presentation. The full set of output files, which includes the DynaMut and mCSM prediction summaries (.doc) as well as the wild-type and mutant structural coordinate files (.pdb and .pse), is provided as Supplementary Materials in the folder named ‘Section S2.7’.

## 3. Results

### 3.1. Nucleotide and Deduced Amino Acid Sequence Analysis of E1 Gene/Protein

**Figure 2:**
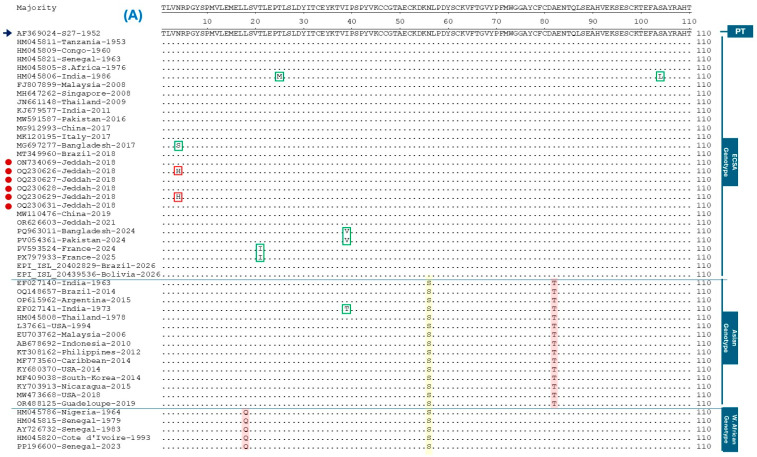

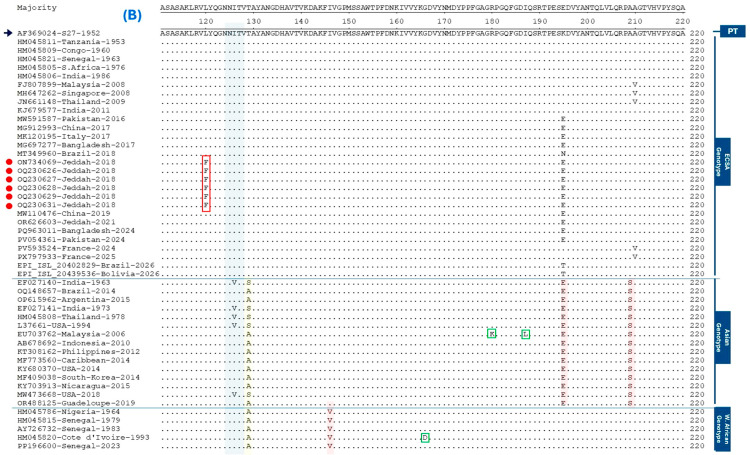

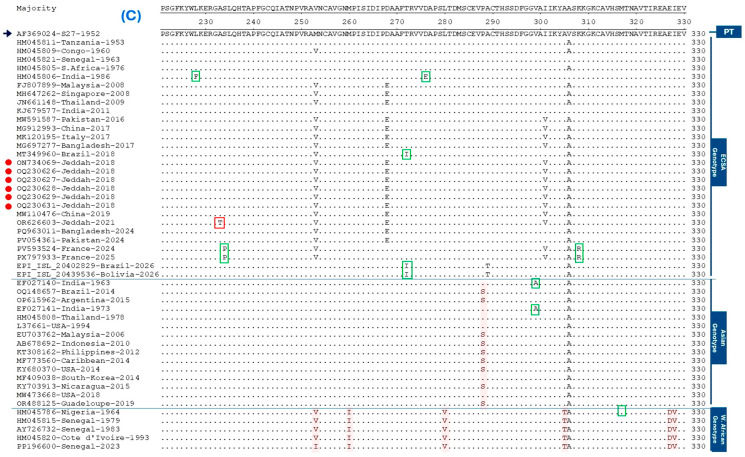

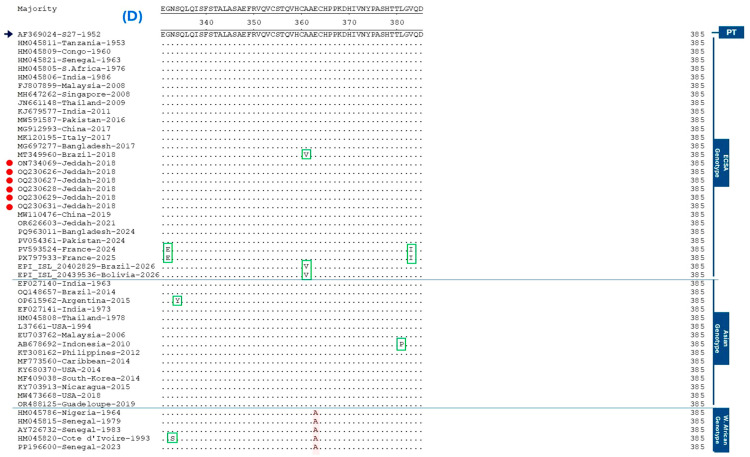
(**A**–**D**) Multiple sequence alignment of the complete *E1* protein sequences of CHIKV from Saudi Arabian isolates and representative international reference strains. The alignment was performed via the ClustalW method within the MegAlign program. The prototype strain (AF369024-S27-1952) served as the reference sequence (indicated by arrow). Dots (.) represent nucleotides identical to the reference. Major genotypes are indicated on the right: ECSA, Asian and West African. Saudi Arabian isolates are highlighted by red circles on the left. Genotype-specific amino acids are highlighted by red rectangles. N-linked glycosylation site is highlighted by green rectangles. Unique amino acids for each strain are shown in green boxes.

Sequence analysis of the CHIKV *E1* gene across the Saudi strains revealed a total of 42 nucleotide point mutations, with corresponding amino acid substitutions distributed among the three major genotypes: ECSA, Asian, and West African. In the ECSA genotype, the Bangladesh 2017 strain (MG697277) harbored N20S, while more recent isolates from France (2024–2025; PV593524, PX797933) and Brazil (2026; EPI-ISL-20384755) exhibited T69I. Historical isolates from India (1986; HM045806) showed T41M and S120L, whereas contemporaneous strains from Bangladesh (2024; PQ963011, PQ963012) and Pakistan (2024; PV054361) carried I55V. The majority of ECSA strains isolated after 2011 displayed a lysine substitution at residue 211, predominantly K211E, with fewer isolates showing K211N or K211T. Residue A226V was observed in older isolates from Mauritius (2006), Malaysia (2008), and Singapore (2008), as well as in recent French isolates (2024–2025). The latter also harbored a cluster of substitutions including S250P, K324R, G348E, and V399I. The Indian isolate HM045806 further presented L244F and D292E. Widespread among ECSA strains were the substitutions M269V, D284E, and I317V (Figure 2C). Brazilian isolates from 2018 (MT349960) and 2026 (EPI_ISL_20384755, EPI_ISL_20402829), alongside Bolivian 2026 isolates, uniquely carried T288I and A377V; however, the 2026 South American isolates additionally shared A305T.

Within the Asian genotype, isolate KJ807897 (Indonesia, 2007) exhibited V28A and N140S, while historical isolates from India (1973; EF027141) carried I55T. Residue V315A was found in both EF027140 (India, 1963) and EF027141. Some Asian strains displayed I142V, and isolate EU703762 (Malaysia, 2006) showed R276K and I203L. More recent Asian genotype isolates from Tonga (2014; LC259088) and Argentina (2015; OP615962) carried M276I and S350Y, respectively. For the West African genotype, limited substitutions were noted: G182D and N349S in the Côte d’Ivoire 1993 isolate (HM045820), and M333L in the Senegal 1966 isolate (HM045816). Three amino acid changes, N20H, L136F (Jeddah strains isolated 2018), and A249T (Jeddah strains isolated 2021), were identified only in the Saudi Arabian strains (Figure 2A–C).

Genotype-specific fixed or highly prevalent changes were also identified. The Asian genotype shared A98T, A225S, and K211E (though the latter also emerged convergently in ECSA). West African strains displayed a distinct set including L34Q, I162V, M269V, M269I, M276I, L296V, L321T, E344D, I345V, and E379A (Figure 2A–D). Shared substitutions between Asian and West African genotypes included N72S, T145S, and T145A. The substitution V322A was found to be common among the majority of strains across all three genotypes, including the Saudi isolates. Regarding glycosylation, prediction algorithms revealed mostly absent or very limited O-linked glycosylation sites in the *E1* protein. The majority of analyzed strains across all genotypes showed no high-confidence O-glycosylation sites. However, a few strains exhibited isolated positive predictions, primarily within the ECSA genotype: PP681186 (Brazil, 2022) with one site at position 194, OM799576 (China, 2020) with two sites at positions 194 and 371, MZ494490 (China, 2018) and OM416162 (China, 2020) with three sites each (positions 218, 222, and 234), and MG697277 (Bangladesh, 2017) with one site at position 4. Additionally, a single positive site at position 218 was detected in one Asian genotype strain (HM045806, India, 1986). In contrast, a single N-linked glycosylation site was consistently identified at residue 141 (Figure 2B), conforming to the canonical motif NITV. This N-linked site was conserved in all Saudi isolates as well as in the majority of ECSA, Asian, and West African strains examined.

### 3.2. Phylogenetic Analysis and Genotype Assignment

**Figure 3:**
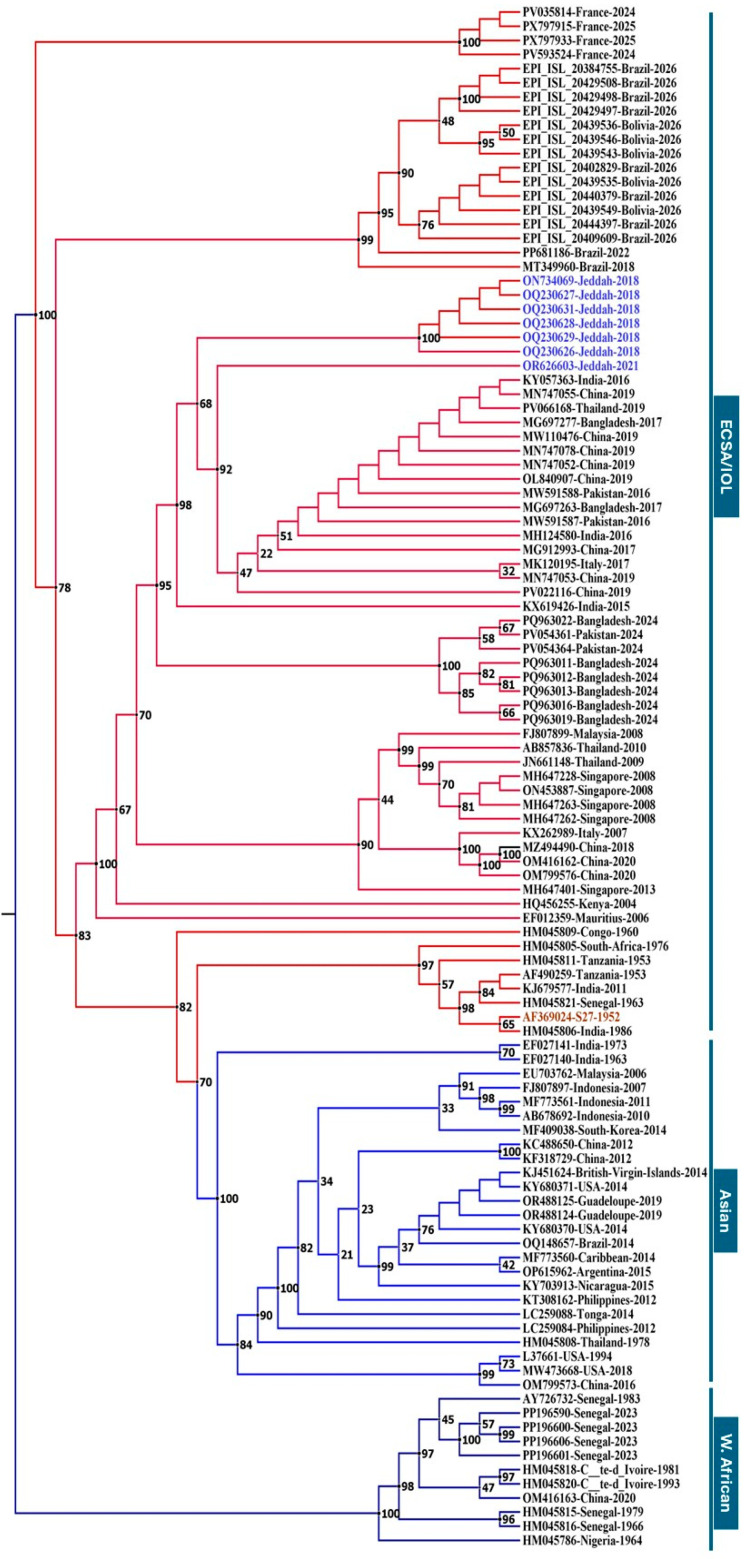
Maximum likelihood phylogenetic tree of CHIKV *E1* gene sequences. The tree was reconstructed using IQ-TREE v3.1.3 under the GTR+I model with 1000 ultrafast bootstrap replicates. Bootstrap support values (≥50%) are shown at internal nodes. Branch lengths are proportional to the number of substitutions per site. The three major genotypes are color-coded: ECSA (red), Asian (blue), and West African (dark blue). Saudi isolates (Jeddah-2018 and Jeddah-2021) are highlighted in orange.

The maximum likelihood phylogenetic tree (Figure 3), reconstructed using IQ-TREE under the GTR+I model with 1000 bootstrap replicates, clearly separated the 109 CHIKV *E1* sequences into three major genotypes, ECSA, Asian, and West African, each forming well-supported monophyletic clades (100% UFBoot support). The ECSA genotype comprised two distinct subclades: the Indian Ocean lineage (IOL), containing recent isolates from France (2024–2025), Brazil (2026), Bolivia (2026), Bangladesh (2024), and Pakistan (2024), and an older group of African and Asian ECSA strains. All seven Saudi isolates (Jeddah-2018 and Jeddah-2021) clustered firmly within the ECSA-IOL subclade with 100% bootstrap support. The 2018 Jeddah isolates (ON734069, OQ230626–OQ230631) grouped closely with East African IOL strains, while the 2021 Jeddah isolate (OR626603) was positioned near strains from the Indian subcontinent and Southeast Asia. This pattern is consistent with multiple independent viral introductions into Saudi Arabia, although sustained local transmission cannot be excluded given the limited sampling. The Asian genotype formed a highly supported clade (100% UFBoot) containing isolates from Thailand, Indonesia, the Caribbean, the USA, China, and Tonga, while the West African genotype (100% UFBoot) comprised isolates from Senegal, Côte d’Ivoire, and Nigeria and served as the outgroup. Short branch lengths among the Saudi isolates indicated recent common ancestry and limited genetic divergence, and the overall topology reflects the global dissemination of the ECSA-IOL lineage since the mid-2000s, with subsequent regional diversification.

### 3.3. Selection Pressure Analysis

To comprehensively characterize the evolutionary constraints acting on the Chikungunya virus *E1* glycoprotein, we employed four complementary codon-based selection detection methods: FEL, FUBAR, MEME, and SLAC (Table 1). Analysis of 385 codons from 109 sequences revealed that the *E1* gene is predominantly under purifying selection, with FUBAR (Figure S5) identifying 140 sites (36.4%) with posterior probability ≥0.9, FEL detecting 125 sites (32.5%) at the reported threshold (*p* < 0.1), and SLAC identifying 58 sites (15.1%) at *p* ≤ 0.1, while MEME does not directly quantify purifying selection but rather detects episodic positive selection. Strong concordance across methods was observed for highly conserved positions, with codons 135 (FEL *p* < 0.001, FUBAR PP = 1.000, SLAC *P*<1 = < 0.001), 307 (FEL *p* < 0.001, FUBAR PP = 1.000, SLAC *P*<1 = 0.002), and 344 (FEL *p* < 0.001, FUBAR PP = 1.000, SLAC *P*<1 = 0.003) exhibiting the strongest evidence of purifying selection, consistent with their critical roles in maintaining *E1* protein structural integrity and fusogenic function. None of the 385 codons met the threshold for robustly supported positive selection. MEME detected isolated episodic signals at codons 99 (*p* = 0.01, β^+^ = 134.01 on ~16% of branches), 306, and 334; however, these lacked corroboration from FEL, SLAC, or FUBAR and therefore represent single-method observations requiring cautious interpretation. Neither FEL nor SLAC identified any statistically significant positive selection sites at their respective thresholds (*p* < 0.05 and *p* ≤ 0.1). Among the positions showing marginal signals in individual analyses, codons 195, 298, and 332 were consistently classified under purifying selection upon multi-method evaluation (codon 195: SLAC P[dN/dS < 1] = 1.000, FUBAR positive PP =0.022; codon 298: FEL *p* = 0.0937, SLAC *P* = 1.000; codon 332: SLAC *P* = 1.000). The predominance of purifying selection (32–36% of sites) across all methods indicates strong functional constraints on the *E1* protein, consistent with its essential role in viral entry and membrane fusion. Although the isolated MEME-only signals at codons 99, 306, and 334 may warrant future functional exploration, they do not meet the stringent corroboration threshold and do not alter the overall conclusion that the *E1* glycoprotein is under strong evolutionary constraint. The complete output files and detailed result tables from each of the four methods are provided as Supplementary Materials in the corresponding folders named “Section S3.4”.

### 3.4. Predicted Stability Effects of Saudi-Unique E1 Mutations

**Figure 4:**
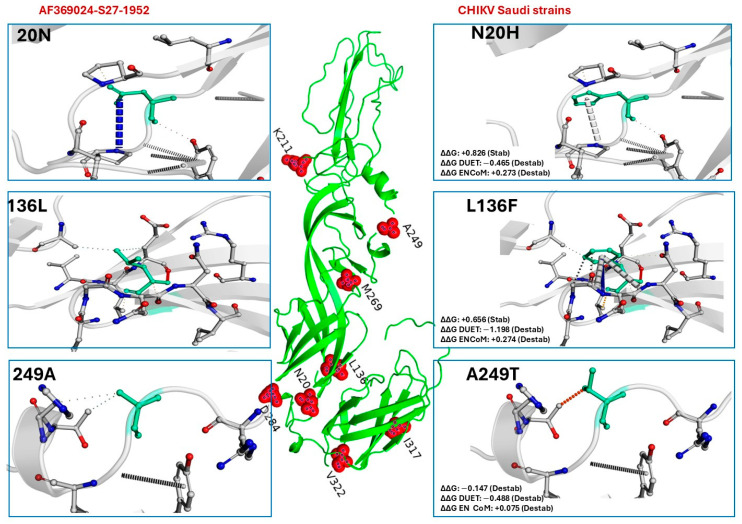
Predicted stability changes in the three Saudi-unique *E1* mutations (N20H, L136F, A249T). For each mutation, the primary DynaMut stability score (ΔΔG, kcal/mol), the consensus structure-based DUET prediction (ΔΔG DUET, kcal/mol), and the normal mode analysis-based ENCoM score (ΔΔG ENCoM, kcal/mol) are shown. Positive values indicate stabilization; negative values indicate destabilization. N20H and L136F were predicted as stabilizing by the primary DynaMut score (+0.826 and +0.656 kcal/mol, respectively) but destabilizing by DUET (−0.465 and −1.198 kcal/mol). A249T was consistently destabilizing (ΔΔG = −0.147 kcal/mol; DUET = −0.488 kcal/mol). ENCoM predicted increased flexibility (destabilizing) for all three mutations. These results indicate that the Saudi-unique mutations are generally destabilizing, with L136F showing the strongest effect (see also Supplementary Figures S1–S4 for the remaining six mutations).

Nine missense substitutions identified in CHIKV *E1* protein from Saudi isolates were analyzed using the DynaMut server, which integrates normal mode analysis (ENCoM) with structure-based (mCSM, SDM) and consensus (DUET) predictions. All mutations are located in loop or irregular secondary structure regions. The primary DynaMut stability score (ΔΔG) predicted five mutations as destabilizing (K211E: −0.482, A249T: −0.147, D284E: −0.355, I317V: −1.200, V322A: −1.225 kcal/mol) and four as stabilizing (N20H: +0.826, L136F: +0.656, K211N: −0.069 (near-neutral), M269V: +0.971 kcal/mol). However, the consensus structure-based predictor DUET predicted destabilizations for eight of the nine mutations, with only D284E being neutral (+0.003 kcal/mol). The strongest destabilizing effects were predicted for V322A (DUET ΔΔG = −2.182 kcal/mol), I317V (−1.241 kcal/mol) and L136F (−1.198 kcal/mol). Normal mode analysis (ENCoM) predicted increased flexibility (destabilizing) for N20H, L136F, A249T, M269V, D284E, I317V and V322A, and decreased flexibility (stabilizing) for K211E and K211N. The three Saudi-unique mutations (N20H, L136F, A249T) showed mixed primary predictions (two stabilizing, one destabilizing) but were all consistently destabilizing by DUET and mCSM, suggesting they are largely destabilizing despite the primary score discrepancies. Overall, the majority of mutations in Saudi strains are predicted to destabilize the *E1* protein, with V322A, I317V and L136F having the most pronounced effects (Figure 4). The predominantly destabilizing nature of these substitutions suggests they may be under purifying selection, consistent with the absence of positive selection signals in the *E1* gene. The discordance between primary DynaMut scores and consensus methods highlights the value of using multiple predictors to assess stability changes. Results for six additional *E1* mutations (K211E, K211N, M269V, D284E, I317V and V322A) are summarized in Supplementary Figures S1–S4, which include their predicted ΔΔG values and interatomic interaction profiles.

## 4. Discussion

The results of this study provide a comprehensive molecular characterization of CHIKV *E1* sequences from Saudi Arabia and highlight the predominant purifying selection shaping the genetic diversity of this essential envelope protein. All Saudi isolates clustered within the ECSA-IOL subclade with strong support. This finding is fully consistent with previous genomic reports indicating that CHIKV in Saudi Arabia belongs to the ECSA lineage and is closely related to contemporaneous strains from East Africa [26]. The 2018 Saudi (Jeddah) isolates grouped closely with East African IOL strains (particularly from Kenya), strengthening the hypothesis of repeated introductions of the IOL from East Africa, presumably driven by travel and population movements [25,27]. In contrast, the 2021 Saudi isolate was associated with a slightly divergent IOL subclade linked to strains from the Indian subcontinent and Southeast Asia [25]. This pattern is suggestive of multiple distinct viral introduction events into Saudi Arabia, although the limited number of sequences (*n* = 7) and restriction to a single gene fragment preclude definitive conclusions regarding the relative contributions of repeated introductions versus sustained local transmission. The Saudi Arabian isolates from 2018 to 2021 formed a distinct, highly supported monophyletic cluster, indicating a relatively recent common ancestor and consistent with localized diversification following introduction(s) into the Arabian Peninsula. The absence of robust temporal signals in the *E1* dataset (root-to-tip regression R^2^ = 1.26 × 10^−3^) precluded precise dating of these introduction events. Nevertheless, the short branch lengths and clustering pattern are consistent with repeated, relatively recent introductions, although sustained local transmission cannot be definitively excluded given the limited sampling.

A key finding of the present study is that the *E1* gene is predominantly under purifying selection. Codon-based analyses using four complementary methods (MEME, FEL, SLAC, and FUBAR) did not identify any site that met the consensus criterion of being significant in at least two methods. FEL identified 125 sites under purifying selection at *p* < 0.1, FUBAR detected 140 sites with posterior probability ≥0.9, and SLAC identified 58 sites at *p* ≤ 0.1, with strong concordance observed at highly conserved positions such as codons 135, 307, and 344 (FEL *p* < 0.001; FUBAR PP = 1.000; SLAC P[dN/dS < 1] = 1.000). Although MEME detected isolated episodic signals at codons 99 (*p* = 0.01, β^+^ = 134.01 on ~16% of branches), 306, and 334, these lacked corroboration from FEL, SLAC, or FUBAR and thus represent single-method observations. Among the positions showing marginal signals in individual analyses, codons 195, 298, and 332 were consistently classified under purifying selection upon multi-method evaluation (codon 195: SLAC P[dN/dS < 1] = 1.000, FUBAR positive PP = 0.022; codon 298: FEL *p* = 0.0937, SLAC *P* = 1.000; codon 332: SLAC *P* = 1.000). This pattern of isolated, method-specific signals is typical for a protein under strong functional constraint, where most nonsynonymous mutations are deleterious and rapidly removed by selection. The overall dN/dS ratio < 1 further supports this conclusion. Our findings are in line with other recent studies of CHIKV *E1* evolution. A whole genome phylogenetic analysis of CHIKV strains from a clustered outbreak in China (2026) demonstrated that the *E1* protein within the Indian Ocean lineage (IOL) genetic background is predominantly subject to purifying selection [23]. Similarly, an analysis of CHIKV during the first epidemic in Colombia also indicated that the *E1* gene is under strong purifying selection [41]. Experimental studies further support this view, demonstrating that mutations affecting *E1*–*E2* heterodimer function are generally deleterious, highlighting the structural and functional constraints acting on these envelope glycoproteins [4]. Thus, despite the rapid global spread of the IOL and its association with major epidemics, the *E1* protein remains evolutionarily constrained, reflecting its essential role in the viral entry process [42].

The three Saudi-unique mutations identified in this study, N20H, L136F and A249T, merit further consideration. Of these, L136F and K211E have been previously documented in Saudi strains isolated in 2018 and 2021 [26]. Notably, the I317V mutation, which was present in our global dataset and has been repeatedly observed in recent CHIKV strains from north central India, Bangladesh and elsewhere [43,44,45], also occurred in the Saudi isolates that we examined. The presence of these shared mutations (K211E, M269V, D284E, I317V, V322A) in Saudi isolates confirms that they belong to the widely circulating IOL subclades that have expanded across Asia and the Indian subcontinent over the past decade [17]. Our DynaMut stability predictions are consistent with this interpretation: mutations such as L136F and I317V were predicted to be destabilizing by the DUET consensus, while many of the other shared mutations also showed either destabilizing or near neutral effects. The consensus structure-based predictor DUET predicted destabilization for eight of the nine mutations examined, with only D284E being neutral (+0.003 kcal/mol). The strongest destabilizing effects were predicted for V322A (DUET ΔΔG = −2.182 kcal/mol), I317V (−1.241 kcal/mol) and L136F (−1.198 kcal/mol). Normal mode analysis (ENCoM) predicted increased flexibility for seven of the nine mutations, further supporting the idea that these substitutions perturb the local dynamics of the protein [40,46]. The continued use of these tools in recent studies [47,48] confirms their reliability for stability assessments. Notably, while the primary DynaMut score for N20H and L136F suggested stabilization, the consensus DUET score predicted destabilization, highlighting the importance of using a multi predictor approach for in silico stability assessments [46].

The widespread occurrence of *E1*-K211E in our Saudi isolates is of particular interest. This mutation has been identified as a marker of the emerging ECSA-IOL subclade that has spread across Malaysia, Thailand, Bangladesh and eastern Africa since 2018 [11,45,49]. Interestingly, experimental studies have shown that the combination of *E1*-K211E and *E2*-V264A can significantly enhance viral fitness in *Aedes aegypti* mosquitoes, suggesting that this dual substitution may contribute to the epidemiological success of this lineage [45,50,51]. None of the Saudi isolates in our dataset carried the canonical A226V mutation, which is known to confer enhanced transmissibility by *Aedes albopictus* [22,52,53]. The absence of this mutation in our analysis is consistent with the observation that the A226V substitution has been largely confined to the IOL strains that circulate in the Indian Ocean region, and its geographic distribution is not uniform [54]. However, the presence of *E1*-K211E may partially compensate in certain vector backgrounds [45]. Future studies should directly assess the phenotypic consequences of the specific combination of mutations present in Saudi strains using reverse genetics and infection assays in both *Ae. aegypti* and *Ae. albopictus*.

The glycosylation analysis revealed a single conserved N-linked glycosylation site at residue 141 (NITV motif), which was present in all Saudi isolates and the vast majority of global sequences. This result is consistent with the known biology of alphavirus *E1* proteins: *E1* contains exactly one N-linked glycosylation site (N141), while *E2* contains two such sites (N263, N345) [55,56]. The glycosylation of *E1*-N141 has been confirmed by crystallographic studies of the *p62*-*E1* heterodimer [57], and N-linked glycans on alphavirus envelope proteins are known to influence protein folding, stability, receptor binding and modulation of host interferon responses [58,59]. The strict conservation of this glycosylation site across all three genotypes and across the entire time span of our dataset underscores its essential role in virus biology. Notably, high-confidence O-linked glycosylation sites were absent in all Saudi isolates and in the vast majority of the analyzed sequences. Only a few non-Saudi ECSA strains showed positive predictions (e.g., positions 210S in PP681186 and OM799576, positions 234S, 238S, 250S in MZ494490 and OM416162). This overall scarcity of O-glycosylation is in agreement with the structural biology of alphavirus envelope proteins, as O-glycans are not commonly described on mature *E1* [58,60].

The present study has several limitations that should be acknowledged. First, the available *E1* sequences from Saudi Arabia are restricted to the Jeddah region and cover a limited time frame (2018–2021). Broader geographical and temporal sampling is needed to capture the full diversity of circulating strains in other provinces, especially the Jazan and Asir regions where Aedes vectors are also present. Second, the selection pressure analyses rely on publicly available sequences, which may be subject to sampling biases; certain genotypes or time periods may be over-represented in the databases. Third, the stability predictions are purely computational and require experimental validation. Although the use of multiple complementary predictors (DynaMut, mCSM, SDM, DUET, ENCoM) increases confidence in the consensus results [40,46], direct measurement of protein stability changes (e.g., by differential scanning fluorimetry or thermal shift assays) would be necessary to confirm the predictions. A further limitation of our phylogenetic inference is the small number of Saudi sequences (*n* = 7) from a single geographic location (Jeddah) and a limited time window (2018–2021), all derived from the *E1* gene fragment rather than whole genomes. Consequently, while the clustering pattern is consistent with repeated introductions, we cannot rule out the possibility that the observed genetic diversity reflects sustained local transmission following one or a few initial introductions. Broader geographic and temporal sampling, combined with whole-genome sequencing, will be required to resolve the relative contributions of these two scenarios.

From a public health perspective, our results highlight the need for continued genomic surveillance of CHIKV in Saudi Arabia. The identification of three unique mutations (N20H, L136F, A249T) together with the presence of globally circulating IOL markers (K211E, I317V, V322A) underscores the fact that the virus is periodically introduced into the Kingdom and that limited local diversification occurs. Although sustained transmission has not yet been documented, the presence of competent *Aedes* vectors in coastal urban centres, the enormous annual influx of pilgrims from endemic regions and the ongoing global expansion of the IOL suggest that the risk of larger outbreaks in the future cannot be discounted [61,62]. Enhanced surveillance, particularly the routine sequencing of *E1* (and ideally whole-genome) from suspected cases, will be essential for the early detection of emergent adaptive variants. In particular, the possible emergence of the *E1* A226V mutation in Saudi isolates would warrant close attention. This substitution is directly responsible for a significant increase in CHIKV infectivity for *Aedes albopictus*, leading to more efficient viral dissemination and transmission [10,53]. Given that Ae. albopictus has already been introduced into the western region of Saudi Arabia [63], and that the Hajj mass gathering continuously imports pathogens from over 190 endemic countries, the potential for the emergence of this adaptive mutation represents a significant public health threat.

## 5. Conclusions

In conclusion, this study provides the first comprehensive molecular characterization of CHIKV *E1* sequences from Saudi Arabia. All seven Saudi isolates belong to the ECSA-IOL subclade, with phylogenetic evidence consistent with multiple introductions from East Africa and the Indian subcontinent, although local circulation cannot be excluded given the limited sampling. The *E1* gene is predominantly under purifying selection, as no codon was robustly identified as positively selected by at least two methods. O-linked glycosylation sites were absent in all Saudi isolates and were rare across the analyzed dataset, with high-confidence predictions limited to only a few non-Saudi ECSA strains. In contrast, a single N-linked glycosylation site at residue 141 was strictly conserved across all strains. Three Saudi-unique mutations (N20H, L136F, A249T) are predicted to be destabilizing, suggesting they are rare, tolerated changes that do not confer an adaptive advantage. The high conservation of the N-linked glycosylation site together with the near-absence of O-glycosylation further highlights the strong functional constraints on the *E1* glycoprotein. Although the risk of emergence of highly adaptive variants appears low at present, continued genomic surveillance remains essential for early detection of any emergent mutations that could alter viral fitness or epidemic potential, particularly during mass gathering events and in high-risk coastal cities.

## Figures and Tables

**Table 1:** Codon-based selection pressure analysis of the CHIKV *E1* protein.

*E1* Codon	MEME (*p*-Value) *	FEL (*p*-Value)	SLAC (P[dN/dS < 1])	FUBAR (Post. Pr.)	Interpretation
13	0.67	0.0016	0.030	0.999	Strong purifying
19	0.67	0.0020	0.019	0.998	Strong purifying
38	0.67	0.0079	0.037	0.990	Strong purifying
45	0.67	0.0090	0.023	0.992	Strong purifying
65	0.67	0.0092	0.051	0.980	Strong purifying
99	0.01	0.3571	0.625	0.400	MEME-only signal
112	0.67	0.0091	0.038	0.990	Strong purifying
135	0.67	<0.001	<0.001	1.000	Strong purifying
143	0.67	0.0008	0.004	0.999	Strong purifying
157	0.67	0.0031	0.012	0.996	Strong purifying
161	0.67	0.0039	0.022	0.992	Strong purifying
194	0.67	0.0009	0.003	0.998	Strong purifying
195	0.16	0.1807	1.000	0.022	Purifying
214	0.67	0.0002	0.003	0.999	Strong purifying
241	0.67	0.0021	0.004	0.998	Strong purifying
254	0.67	0.0004	0.003	0.999	Strong purifying
278	0.67	0.0126	0.105	0.993	Purifying
298	0.12	0.0937	1.000	0.187	Purifying
306	0.06	0.8383	0.704	0.550	MEME-only signal
307	0.67	<0.001	0.002	1.000	Strong purifying
318	0.67	0.0037	0.012	0.996	Strong purifying
332	0.12	0.1000	1.000	0.201	Purifying
334	0.05	0.7646	0.556	0.674	MEME-only signal
344	0.67	<0.001	0.003	1.000	Strong purifying
365	0.67	0.0009	0.010	0.998	Strong purifying

* Abbreviations: FEL, Fixed Effects Likelihood; FUBAR, Fast Unconstrained Bayesian Approximation; MEME, Mixed Effects Model of Evolution; SLAC, Single-Likelihood Ancestral Counting. The “Interpretation” column synthesizes evidence across methods: “Strong purifying” indicates consistent signals of negative selection across multiple methods; “Pervasive positive” indicates evidence of positive selection across most branches (FUBAR).

## Data Availability

All raw and processed data supporting the findings are provided as Supplementary Folders. Sequence metadata are available in the folder named “Section S2.2—Table S1”. The output tables and .JSON files from the codon-based selection pressure analyses (FUBAR, FEL, MEME, and SLAC) are in the folder named “Section S3.4 (FUBAR, FEL, MEME, SLAC)”. Computational mutagenesis stability predictions from DynaMut, DUET, and mCSM, along with the corresponding structural coordinate files are in the folder “Section S2.7 (DynMut, DUET and mCSM Analysis)”. Supplementary Figures S1–S4 are organized into the folder “Section S3.4—Figures S5” and the folder “Section S3.5—Figures S1–S4”. Additional inquiries may be directed to the corresponding author.

## Supplementary Materials

The following supporting information can be downloaded at: https://www.mdpi.com/article/10.3390/v18070791/s1.

## Abbreviations

The following abbreviations are used in this manuscript:

CHIKV	Chikungunya virus
*E1*	Envelope glycoprotein 1 (class II fusion protein)
ECSA	East/Central/South African (genotype)
IOL	Indian Ocean lineage (sub-lineage of ECSA)
MEME	Mixed Effects Model of Evolution (detects episodic diversifying selection)
FEL	Fixed Effects Likelihood (detects pervasive selection)
SLAC	Single-Likelihood Ancestor Counting (detects pervasive selection)
FUBAR	Fast Unconstrained Bayesian AppRoximation (Bayesian estimate of pervasive selection)
BEAST	Bayesian Evolutionary Analysis Sampling Trees (software for divergence time analysis)
MCMC	Markov chain Monte Carlo (sampling method used in BEAST)
HPD	Highest Posterior Density (95% confidence interval for divergence time)
PP	Posterior probability (clade support in Bayesian tree)
dN/dS	Ratio of non-synonymous to synonymous substitutions (measure of selection pressure)
ΔΔG	Change in Gibbs free energy (kcal/mol; negative = destabilizing mutation)
DUET	Consensus structure-based predictor for mutation stability effects
ENCoM	Normal mode analysis-based stability score (part of DynaMut)
mCSM	Structure-based stability predictor (used in DUET consensus)
SDM	Structure-based stability predictor (used in DUET consensus)
NMA	Normal mode analysis (used to predict flexibility changes)
