# Supplementary material for: Molecular Evolution of the Chikungunya Virus E1 Gene in Saudi Arabia: Predominance of Purifying Selection and ECSA/IOL Lineage Circulation
Source: Viruses. 2026 Jul 19;18(7):791. doi: 10.3390/v18070791 (PMC13431504; doi:10.3390/v18070791)
Supplement: Supplementary file 1 [file viruses-18-00791-s001.zip › Section S2.2. - Table S1.zip!/Table S1.pdf]

Table S1. List of CHIKV Strains used in sequence and phylogenetic analysis

No.	Accession Number	Country	Year	Genotype
1	PV035814	France	2024	ECSA/IOL
2	PX797915	France	2025	ECSA/IOL
3	PX797933	France	2025	ECSA/IOL
4	PV593524	France	2024	ECSA/IOL
5	EPI__ISL_20384755	Brazil	2026	ECSA/IOL
6	EPI_ ISL_20429508	Brazil	2026	ECSA/IOL
7	EPI_ ISL_20429498	Brazil	2026	ECSA/IOL
8	EPI_ ISL_20429497	Brazil	2026	ECSA/IOL
9	EPI_ ISL_20439536	Bolivia	2026	ECSA/IOL
10	EPI_ ISL_20439546	Bolivia	2026	ECSA/IOL
11	EPI_ ISL_20439543	Bolivia	2026	ECSA/IOL
12	EPI_ ISL_20402829	Brazil	2026	ECSA/IOL
13	EPI_ ISL_20439535	Bolivia	2026	ECSA/IOL
14	EPI_ ISL_20440379	Brazil	2026	ECSA/IOL
15	EPI_ ISL_20439549	Bolivia	2026	ECSA/IOL
16	EPI_ ISL_20444397	Brazil	2026	ECSA/IOL
17	EPI_ ISL_20409609	Brazil	2026	ECSA/IOL
18	PP681186	Brazil	2022	ECSA/IOL
19	MT349960	Brazil	2018	ECSA/IOL
20	ON734069	Jeddah	2018	ECSA/IOL

No.	Accession Number	Country	Year	Genotype
21	OQ230627	Jeddah	2018	ECSA/IOL
22	OQ230631	Jeddah	2018	ECSA/IOL
23	OQ230628	Jeddah	2018	ECSA/IOL
24	OQ230629	Jeddah	2018	ECSA/IOL
25	OQ230626	Jeddah	2018	ECSA/IOL
26	OR626603	Jeddah	2021	ECSA/IOL
27	KY057363	India	2016	ECSA/IOL
28	MN747055	China	2019	ECSA/IOL
29	PV066168	Thailand	2019	ECSA/IOL
30	MG697277	Bangladesh	2017	ECSA/IOL
31	MW110476	China	2019	ECSA/IOL
32	MN747078	China	2019	ECSA/IOL
33	MN747052	China	2019	ECSA/IOL
34	OL840907	China	2019	ECSA/IOL
35	MW591588	Pakistan	2016	ECSA/IOL
36	MG697263	Bangladesh	2017	ECSA/IOL
37	MW591587	Pakistan	2016	ECSA/IOL
38	MH124580	India	2016	ECSA/IOL
39	MG912993	China	2017	ECSA/IOL
40	MK120195	Italy	2017	ECSA/IOL
41	MN747053	China	2019	ECSA/IOL

No.	Accession Number	Country	Year	Genotype
42	PV022116	China	2019	ECSA/IOL
43	KX619426	India	2015	ECSA/IOL
44	PQ963022	Bangladesh	2024	ECSA/IOL
45	PV054361	Pakistan	2024	ECSA/IOL
46	PV054364	Pakistan	2024	ECSA/IOL
47	PQ963011	Bangladesh	2024	ECSA/IOL
48	PQ963012	Bangladesh	2024	ECSA/IOL
49	PQ963013	Bangladesh	2024	ECSA/IOL
50	PQ963016	Bangladesh	2024	ECSA/IOL
51	PQ963019	Bangladesh	2024	ECSA/IOL
52	FJ807899	Malaysia	2008	ECSA/IOL
53	AB857836	Thailand	2010	ECSA/IOL
54	JN661148	Thailand	2009	ECSA/IOL
55	MH647228	Singapore	2008	ECSA/IOL
56	ON453887	Singapore	2008	ECSA/IOL
57	MH647263	Singapore	2008	ECSA/IOL
58	MH647262	Singapore	2008	ECSA/IOL
59	KX262989	Italy	2007	ECSA/IOL
60	MZ494490	China	2018	ECSA/IOL
61	OM416162	China	2020	ECSA/IOL
62	OM799576	China	2020	ECSA/IOL

No.	Accession Number	Country	Year	Genotype
63	MH647401	Singapore	2013	ECSA/IOL
64	HQ456255	Kenya	2004	ECSA/IOL
65	EF012359	Mauritius	2006	ECSA/IOL
66	HM045809	Congo	1960	ECSA/IOL
67	HM045805	South Africa	1976	ECSA/IOL
68	HM045811	Tanzania	1953	ECSA/IOL
69	KJ679577	India	2011	ECSA/IOL
70	HM045821	Senegal	1963	ECSA/IOL
71	AF369024	S27	1952	ECSA/IOL
72	HM045806	India	1986	ECSA/IOL
73	AF490259	Tanzania	1953	ECSA/IOL
74	EF027141	India	1973	Asian
75	EF027140	India	1963	Asian
76	EU703762	Malaysia	2006	Asian
77	FJ807897	Indonesia	2007	Asian
78	MF773561	Indonesia	2011	Asian
79	AB678692	Indonesia	2010	Asian
80	MF409038	South Korea	2014	Asian
81	KC488650	China	2012	Asian
82	KF318729	China	2012	Asian
83	KJ451624	British Virgin Isl.	2014	Asian

No.	Accession Number	Country	Year	Genotype
84	KY680371	USA	2014	Asian
85	OR488125	Guadeloupe	2019	Asian
86	OR488124	Guadeloupe	2019	Asian
87	KY680370	USA	2014	Asian
88	OQ148657	Brazil	2014	Asian
89	MF773560	Caribbean	2014	Asian
90	OP615962	Argentina	2015	Asian
91	KY703913	Nicaragua	2015	Asian
92	KT308162	Philippines	2012	Asian
93	LC259088	Tonga	2014	Asian
94	LC259084	Philippines	2012	Asian
95	HM045808	Thailand	1978	Asian
96	L37661	USA	1994	Asian
97	MW473668	USA	2018	Asian
98	OM799573	China	2016	Asian
99	AY726732	Senegal	1983	W. African
100	PP196590	Senegal	2023	W. African
101	PP196600	Senegal	2023	W. African
102	PP196606	Senegal	2023	W. African
103	PP196601	Senegal	2023	W. African
104	HM1045818	Côte d'Ivoire	1981	W. African

No.	Accession Number	Country	Year	Genotype
105	HM1045820	Côte d'Ivoire	1993	W. African
106	OM416163	China	2020	W. African
107	HM1045815	Senegal	1979	W. African
108	HM1045816	Senegal	1966	W. African
109	HM1045786	Nigeria	1964	W. African
