# Supplementary material for: Molecular Evolution of the Chikungunya Virus E1 Gene in Saudi Arabia: Predominance of Purifying Selection and ECSA/IOL Lineage Circulation
Source: Viruses. 2026 Jul 19;18(7):791. doi: 10.3390/v18070791 (PMC13431504; doi:10.3390/v18070791)
Supplement: Supplementary file 1 [file viruses-18-00791-s001.zip › Section S2.7. (DynMut,DUET and mCSM Analysis).zip!/Dynamut Analysis.pdf]

Dynamut Analysis

N20H

Prediction Outcome

ΔΔG: 0.826 kcal/mol (Stabilizing)

NMA Based Predictions

ΔΔG ENCoM: 0.273 kcal/mol (Destabilizing)

Other Structure-Based Predictions

ΔΔG mCSM: -0.795 kcal/mol (Destabilizing)

ΔΔG SDM: 0.840 kcal/mol (Stabilizing)

ΔΔG DUET: -0.465 kcal/mol (Destabilizing)

L136F

Prediction Outcome

ΔΔG: 0.656 kcal/mol (Stabilizing)

NMA Based Predictions

ΔΔG ENCoM: 0.274 kcal/mol (Destabilizing)

Other Structure-Based Predictions

ΔΔG mCSM: -1.413 kcal/mol (Destabilizing)

ΔΔG SDM: -0.100 kcal/mol (Destabilizing)

ΔΔG DUET: -1.198 kcal/mol (Destabilizing)

Δ Vibrational Entropy Energy Between Wild-Type and Mutant

$\Delta\Delta S_{Vib}$ ENCoM: -0.343 $kcal.mol^{-1}.K^{-1}$ (Decrease of molecule flexibility)

K211E

Prediction Outcome

ΔΔG: -0.482 kcal/mol (Destabilizing)

NMA Based Predictions

ΔΔG ENCoM: -0.013 kcal/mol (Destabilizing)

Other Structure-Based Predictions

ΔΔG mCSM: -0.466 kcal/mol (Destabilizing)

ΔΔG SDM: 0.280 kcal/mol (Stabilizing)

ΔΔG DUET: -0.098 kcal/mol (Destabilizing)

Δ Vibrational Entropy Energy Between Wild-Type and Mutant

$\Delta\Delta S_{Vib}$ ENCoM: 0.016 $kcal.mol^{-1}.K^{-1}$ (Increase of molecule flexibility)

K211N

Prediction Outcome

ΔΔG: -0.069 kcal/mol (Destabilizing)

NMA Based Predictions

ΔΔG ENCoM: -0.023 kcal/mol (Destabilizing)

Other Structure-Based Predictions

ΔΔG mCSM: -0.176 kcal/mol (Destabilizing)

ΔΔG SDM: 0.140 kcal/mol (Stabilizing)

ΔΔG DUET: 0.083 kcal/mol (Stabilizing)

Δ Vibrational Entropy Energy Between Wild-Type and Mutant

$\Delta\Delta S_{Vib}$ ENCoM: 0.029 kcal.mol^{-1}.K^{-1} (Increase of molecule flexibility)

A249T

Prediction Outcome

ΔΔG: -0.147 kcal/mol (Destabilizing)

NMA Based Predictions

ΔΔG ENCoM: 0.075 kcal/mol (Destabilizing)

Other Structure-Based Predictions

ΔΔG mCSM: -0.689 kcal/mol (Destabilizing)

ΔΔG SDM: -0.760 kcal/mol (Destabilizing)

ΔΔG DUET: -0.488 kcal/mol (Destabilizing)

M269V

Prediction Outcome

ΔΔG: 0.971 kcal/mol (Stabilizing)

NMA Based Predictions

ΔΔG ENCoM: 0.129 kcal/mol (Destabilizing)

Other Structure-Based Predictions

ΔΔG mCSM: -0.988 kcal/mol (Destabilizing)

ΔΔG SDM: 1.390 kcal/mol (Stabilizing)

ΔΔG DUET: -0.132 kcal/mol (Destabilizing)

Δ Vibrational Entropy Energy Between Wild-Type and Mutant

$\Delta\Delta S_{Vib}$ ENCoM: -0.161 kcal.mol^{-1}.K^{-1} (Decrease of molecule flexibility)

D284E

Prediction Outcome

ΔΔG: -0.355 kcal/mol (Destabilizing)

NMA Based Predictions

ΔΔG ENCoM: -0.223 kcal/mol (Destabilizing)

Other Structure-Based Predictions

ΔΔG mCSM: -0.478 kcal/mol (Destabilizing)

ΔΔG SDM: 0.400 kcal/mol (Stabilizing)

ΔΔG DUET: 0.003 kcal/mol (Stabilizing)

Δ Vibrational Entropy Energy Between Wild-Type and Mutant

$\Delta\Delta S_{Vib}$ ENCoM: 0.278 kcal.mol^{-1}.K^{-1} (Increase of molecule flexibility)

I317V

Prediction Outcome

ΔΔG: -1.200 kcal/mol (Destabilizing)

NMA Based Predictions

ΔΔG ENCoM: -0.013 kcal/mol (Destabilizing)

Other Structure-Based Predictions

ΔΔG mCSM: -1.630 kcal/mol (Destabilizing)

ΔΔG SDM: 0.290 kcal/mol (Stabilizing)

ΔΔG DUET: -1.241 kcal/mol (Destabilizing)

Δ Vibrational Entropy Energy Between Wild-Type and Mutant

$\Delta\Delta S_{Vib}$ ENCoM: 0.017 $kcal.mol^{-1}.K^{-1}$ (Increase of molecule flexibility)

V322A

Prediction Outcome

ΔΔG: -1.225 kcal/mol (Destabilizing)

NMA Based Predictions

ΔΔG ENCoM: -0.076 kcal/mol (Destabilizing)

Other Structure-Based Predictions

ΔΔG mCSM: -1.783 kcal/mol (Destabilizing)

ΔΔG SDM: -2.660 kcal/mol (Destabilizing)

ΔΔG DUET: -2.182 kcal/mol (Destabilizing)

Δ Vibrational Entropy Energy Between Wild-Type and Mutant

$\Delta\Delta S_{Vib}$ ENCoM: 0.095 $kcal.mol^{-1}.K^{-1}$ (Increase of molecule flexibility)
