# Supplementary material for: Molecular Evolution of the Chikungunya Virus E1 Gene in Saudi Arabia: Predominance of Purifying Selection and ECSA/IOL Lineage Circulation
Source: Viruses. 2026 Jul 19;18(7):791. doi: 10.3390/v18070791 (PMC13431504; doi:10.3390/v18070791)
Supplement: Supplementary file 1 [file viruses-18-00791-s001.zip › Section S2.7. (DynMut,DUET and mCSM Analysis).zip!/mCSM Predicted Stability Change.pdf]

1. **mCSM Predicted Stability Change (ΔΔG):**

   -0.728 kcal/mol (Destabilizing)

   SDM Predicted Stability Change (ΔΔG):

   0.82 kcal/mol (Stabilizing)

   DUET Predicted Stability Change (ΔΔG):

   -0.408 kcal/mol (Destabilizing)

   Mutation:

   Wild-type: ASN

   Position: 20

   Mutant-type: HIS

   Chain: A

   Secondary structure: Loop or irregular

2. **mCSM Predicted Stability Change (ΔΔG):**

   -0.534 kcal/mol (Destabilizing)

   SDM Predicted Stability Change (ΔΔG):

   -0.15 kcal/mol (Destabilizing)

   DUET Predicted Stability Change (ΔΔG):

   -0.211 kcal/mol (Destabilizing)

   Mutation:

   Wild-type: ALA

   Position: 249

   Mutant-type: THR

   Chain: A

   Secondary structure: Loop or irregular

3. **mCSM Predicted Stability Change (ΔΔG):**

   -1.024 kcal/mol (Destabilizing)

   SDM Predicted Stability Change (ΔΔG):

   1.23 kcal/mol (Stabilizing)

   DUET Predicted Stability Change (ΔΔG):

   -0.319 kcal/mol (Destabilizing)

   Mutation:

   Wild-type: MET

   Position: 269

   Mutant-type: VAL

   Chain: A

   Secondary structure: Loop or irregular

4. **mCSM Predicted Stability Change (ΔΔG):**

   -0.477 kcal/mol (Destabilizing)

   SDM Predicted Stability Change (ΔΔG):

   0.02 kcal/mol (Stabilizing)

   DUET Predicted Stability Change (ΔΔG):

   -0.158 kcal/mol (Destabilizing)

   Mutation:

   Wild-type: ASP

   Position: 284

   Mutant-type: GLU

   Chain: A

   Secondary structure: Loop or irregular

5. **mCSM Predicted Stability Change (ΔΔG):**

   -1.381 kcal/mol (Destabilizing)

   SDM Predicted Stability Change (ΔΔG):

   0.29 kcal/mol (Stabilizing)

   DUET Predicted Stability Change (ΔΔG):

   -1.055 kcal/mol (Destabilizing)

   Mutation:

   Wild-type: ILE

   Position: 317

   Mutant-type: VAL

   Chain: A

   Secondary structure: Loop or irregular

6. **mCSM Predicted Stability Change (ΔΔG):**

   -1.783 kcal/mol (Destabilizing)

   SDM Predicted Stability Change (ΔΔG):

   -2.66 kcal/mol (Destabilizing)

   DUET Predicted Stability Change (ΔΔG):

   -2.202 kcal/mol (Destabilizing)

   Mutation:

   Wild-type: VAL

   Position: 322

   Mutant-type: ALA

   Chain: A

   Secondary structure: Loop or irregular

7. **mCSM Predicted Stability Change (ΔΔG):**

   -0.466 kcal/mol (Destabilizing)

   SDM Predicted Stability Change (ΔΔG):

   0.28 kcal/mol (Stabilizing)

   DUET Predicted Stability Change (ΔΔG):

   -0.115 kcal/mol (Destabilizing)

   Mutation:

   Wild-type: LYS

   Position: 211

   Mutant-type: GLU

   Chain: A

   Secondary structure: Loop or irregular

8. **mCSM Predicted Stability Change (ΔΔG):**

   -0.176 kcal/mol (Destabilizing)

   SDM Predicted Stability Change (ΔΔG):

   0.14 kcal/mol (Stabilizing)

   DUET Predicted Stability Change (ΔΔG):

   0.076 kcal/mol (Stabilizing)

   Mutation:

   Wild-type: LYS

   Position: 211

   Mutant-type: ASN

   Chain: A

   Secondary structure: Loop or irregular

9. **mCSM Predicted Stability Change (ΔΔG):**

   -1.408 kcal/mol (Destabilizing)

   SDM Predicted Stability Change (ΔΔG):

   -0.1 kcal/mol (Destabilizing)

   DUET Predicted Stability Change (ΔΔG):

   -1.28 kcal/mol (Destabilizing)

   Mutation:

   Wild-type: LEU

   Position: 136

   Mutant-type: PHE

   Chain: A

   Secondary structure: Loop or irregular
