# Supplementary material for: Molecular Evolution of the Chikungunya Virus E1 Gene in Saudi Arabia: Predominance of Purifying Selection and ECSA/IOL Lineage Circulation
Source: Viruses. 2026 Jul 19;18(7):791. doi: 10.3390/v18070791 (PMC13431504; doi:10.3390/v18070791)
Supplement: Supplementary file 1 [file viruses-18-00791-s001.zip › Section S3.4 (FUBAR, FEL, MEME, SLAC).zip!/FEL.zip!/FEL Table.pdf]

Table. Detailed site-by-site results from the FEL analysis

codon	alpha	beta	alpha=beta	LRT	p-value	Total branch length	▲ class
1	0.000	0.000	0.000	0.000	1.0000	0.000	Invariable
2	0.431	0.000	0.167	1.873	0.1712	0.262	Neutral
3	0.000	0.000	0.000	0.000	1.0000	0.000	Invariable
4	3.618	1.691	2.091	0.812	0.3674	3.283	Neutral
5	0.000	0.000	0.000	0.000	1.0000	0.000	Invariable
6	0.628	0.242	0.349	0.422	0.5158	0.548	Neutral
7	0.555	0.766	0.682	0.068	0.7939	1.072	Neutral
8	1.393	0.000	0.292	2.914	0.0878	0.459	Purifying
9	1.459	0.000	0.214	3.653	0.0560	0.335	Purifying
10	0.646	0.249	0.358	0.423	0.5154	0.562	Neutral
11	0.000	0.000	0.000	0.000	1.0000	0.000	Invariable
12	2.006	0.620	0.968	1.227	0.2680	1.520	Neutral
13	3.068	0.000	1.056	10.006	0.0016	1.659	Purifying
14	0.000	0.000	0.000	0.000	1.0000	0.000	Invariable
15	0.000	0.000	0.000	0.000	1.0000	0.000	Invariable
16	1.456	1.503	1.491	0.001	0.9750	2.341	Neutral
17	0.870	0.000	0.346	3.626	0.0569	0.543	Purifying
18	0.461	0.328	0.385	0.053	0.8179	0.605	Neutral
19	3.783	0.000	1.073	9.587	0.0020	1.685	Purifying
20	0.000	0.000	0.000	0.000	1.0000	0.000	Invariable
21	2.148	0.623	1.192	2.051	0.1521	1.872	Neutral
22	0.834	0.303	0.444	0.480	0.4884	0.697	Neutral
23	3.641	0.345	0.885	3.647	0.0562	1.389	Purifying
24	0.000	0.517	0.393	1.085	0.2977	0.616	Neutral
25	3.640	0.303	1.164	6.185	0.0129	1.828	Purifying
26	1.908	0.310	0.941	3.135	0.0766	1.477	Purifying
27	0.718	0.341	0.467	0.257	0.6120	0.734	Neutral
28	0.437	0.000	0.147	2.169	0.1408	0.230	Neutral

29	0.681	0.350	0.461	0.219	0.6401	0.724	Neutral
30	0.000	0.389	0.291	0.581	0.4459	0.458	Neutral
31	0.000	0.000	0.000	0.000	1.0000	0.000	Invariable
32	1.468	0.301	0.644	1.701	0.1921	1.012	Neutral
33	1.287	0.000	0.221	3.348	0.0673	0.347	Purifying
34	1.609	0.000	0.271	3.478	0.0622	0.426	Purifying
35	1.251	0.782	0.895	0.129	0.7191	1.405	Neutral
36	0.000	0.772	0.625	0.835	0.3609	0.981	Neutral
37	1.171	0.000	0.395	4.252	0.0392	0.620	Purifying
38	2.242	0.000	0.627	7.064	0.0079	0.984	Purifying
39	0.000	0.477	0.379	0.918	0.3380	0.595	Neutral
40	1.412	1.117	1.201	0.066	0.7979	1.886	Neutral
41	1.494	0.000	0.569	5.713	0.0168	0.894	Purifying
42	0.000	0.000	0.000	0.000	1.0000	0.000	Invariable
43	0.000	0.000	0.000	0.000	1.0000	0.000	Invariable
44	0.000	0.000	0.000	0.000	1.0000	0.000	Invariable
45	6.125	0.343	1.202	6.819	0.0090	1.888	Purifying
46	0.000	0.000	0.000	0.000	1.0000	0.000	Invariable
47	2.139	0.000	0.440	5.967	0.0146	0.691	Purifying
48	0.439	0.376	0.404	0.012	0.9132	0.635	Neutral
49	0.000	0.000	0.000	0.000	1.0000	0.000	Invariable
50	0.856	0.000	0.244	2.504	0.1135	0.382	Neutral
51	0.000	0.334	0.272	0.406	0.5241	0.427	Neutral
52	3.347	0.000	0.453	7.174	0.0074	0.711	Purifying
53	0.000	0.340	0.274	0.406	0.5240	0.431	Neutral
54	0.000	0.332	0.258	0.506	0.4770	0.405	Neutral
55	2.859	0.365	0.697	2.052	0.1520	1.094	Neutral
56	1.214	1.019	1.058	0.019	0.8898	1.661	Neutral
57	0.411	0.000	0.174	1.696	0.1928	0.274	Neutral
58	1.067	0.505	0.689	0.521	0.4704	1.082	Neutral
59	1.278	0.338	0.534	0.775	0.3786	0.839	Neutral

60	0.000	0.000	0.000	0.000	1.0000	0.000	Invariable
61	1.365	0.000	0.211	3.543	0.0598	0.331	Purifying
62	0.689	0.000	0.210	2.346	0.1256	0.330	Neutral
63	0.000	0.000	0.000	0.000	1.0000	0.000	Invariable
64	0.000	0.300	0.200	0.807	0.3691	0.313	Neutral
65	2.136	0.000	0.369	6.781	0.0092	0.579	Purifying
66	0.559	0.307	0.395	0.172	0.6786	0.621	Neutral
67	0.872	0.770	0.802	0.010	0.9185	1.258	Neutral
68	0.000	0.000	0.000	0.000	1.0000	0.000	Invariable
69	0.000	0.000	0.000	0.000	1.0000	0.000	Invariable
70	0.000	0.247	0.190	0.519	0.4712	0.299	Neutral
71	0.691	0.486	0.538	0.075	0.7848	0.844	Neutral
72	0.000	0.000	0.000	0.000	1.0000	0.000	Invariable
73	0.008	0.644	0.633	0.066	0.7977	0.995	Neutral
74	0.000	0.759	0.466	1.911	0.1669	0.732	Neutral
75	1.418	0.000	0.473	4.127	0.0422	0.743	Purifying
76	0.629	0.000	0.219	2.074	0.1498	0.343	Neutral
77	3.434	0.000	0.596	6.127	0.0133	0.936	Purifying
78	0.000	0.000	0.000	0.000	1.0000	0.000	Invariable
79	0.954	0.483	0.579	0.261	0.6094	0.909	Neutral
80	0.000	0.332	0.256	0.525	0.4689	0.402	Neutral
81	0.000	0.000	0.000	0.000	1.0000	0.000	Invariable
82	1.551	0.344	0.836	1.892	0.1690	1.313	Neutral
83	0.000	0.000	0.000	0.000	1.0000	0.000	Invariable
84	2.596	0.649	1.078	1.790	0.1809	1.692	Neutral
85	0.000	0.000	0.000	0.000	1.0000	0.000	Invariable
86	1.843	0.000	0.292	3.588	0.0582	0.458	Purifying
87	0.663	0.000	0.219	2.192	0.1387	0.344	Neutral
88	1.353	0.000	0.210	3.528	0.0603	0.330	Purifying
89	1.773	0.357	0.601	1.102	0.2938	0.944	Neutral
90	0.000	0.000	0.000	0.000	1.0000	0.000	Invariable

91	1.428	0.253	0.550	2.044	0.1528	0.863	Neutral
92	1.500	0.000	0.430	4.925	0.0265	0.675	Purifying
93	0.000	0.334	0.271	0.405	0.5244	0.426	Neutral
94	1.619	0.000	0.286	3.423	0.0643	0.449	Purifying
95	1.161	0.000	0.396	4.217	0.0400	0.622	Purifying
96	2.830	0.364	0.653	1.790	0.1809	1.025	Neutral
97	0.713	0.000	0.236	2.217	0.1365	0.370	Neutral
98	0.011	0.275	0.223	0.405	0.5244	0.350	Neutral
99	0.000	0.778	0.629	0.848	0.3571	0.987	Neutral
100	0.000	0.629	0.455	1.269	0.2599	0.715	Neutral
101	1.773	0.357	0.601	1.102	0.2938	0.944	Neutral
102	0.628	0.235	0.340	0.448	0.5031	0.534	Neutral
103	0.000	0.000	0.000	0.000	1.0000	0.000	Invariable
104	0.799	0.359	0.511	0.292	0.5886	0.803	Neutral
105	2.019	0.000	0.532	5.552	0.0185	0.835	Purifying
106	4.638	0.000	0.654	7.361	0.0067	1.026	Purifying
107	1.377	0.865	0.953	0.136	0.7126	1.496	Neutral
108	1.491	0.000	0.611	5.278	0.0216	0.959	Purifying
109	1.472	0.000	0.364	5.483	0.0192	0.572	Purifying
110	1.499	0.000	0.419	4.811	0.0283	0.658	Purifying
111	0.000	0.000	0.000	0.000	1.0000	0.000	Invariable
112	2.079	0.000	0.641	6.802	0.0091	1.006	Purifying
113	1.688	0.863	1.138	0.424	0.5149	1.787	Neutral
114	1.711	0.000	0.511	4.757	0.0292	0.802	Purifying
115	0.839	0.000	0.291	2.796	0.0945	0.457	Purifying
116	0.000	0.000	0.000	0.000	1.0000	0.000	Invariable
117	0.623	0.000	0.161	2.689	0.1011	0.253	Neutral
118	0.000	0.000	0.000	0.000	1.0000	0.000	Invariable
119	0.633	0.000	0.201	2.248	0.1338	0.316	Neutral
120	0.435	0.221	0.294	0.227	0.6335	0.461	Neutral
121	0.000	0.000	0.000	0.000	1.0000	0.000	Invariable

122	0.000	0.350	0.312	0.242	0.6231	0.489	Neutral
123	0.872	0.380	0.531	0.312	0.5762	0.834	Neutral
124	1.410	0.975	1.113	0.150	0.6985	1.747	Neutral
125	1.232	0.000	0.235	3.212	0.0731	0.369	Purifying
126	1.690	0.250	0.587	2.444	0.1180	0.922	Neutral
127	0.981	0.000	0.369	3.797	0.0514	0.580	Purifying
128	1.815	0.000	0.451	5.433	0.0198	0.708	Purifying
129	0.437	1.000	0.741	0.524	0.4690	1.163	Neutral
130	1.321	0.000	0.448	4.231	0.0397	0.703	Purifying
131	0.691	0.000	0.246	2.035	0.1537	0.386	Neutral
132	2.367	0.342	0.964	3.152	0.0758	1.514	Purifying
133	1.287	0.291	0.468	0.920	0.3375	0.734	Neutral
134	0.643	0.375	0.468	0.139	0.7090	0.736	Neutral
135	23.109	0.000	1.753	24.277	0.0000	2.752	Purifying
136	1.291	0.000	0.350	5.015	0.0251	0.549	Purifying
137	0.000	0.000	0.000	0.000	1.0000	0.000	Invariable
138	0.633	0.000	0.202	2.249	0.1337	0.316	Neutral
139	0.000	0.000	0.000	0.000	1.0000	0.000	Invariable
140	1.095	0.299	0.583	1.150	0.2836	0.915	Neutral
141	0.000	0.000	0.000	0.000	1.0000	0.000	Invariable
142	0.000	0.332	0.257	0.505	0.4773	0.404	Neutral
143	4.076	0.000	1.200	11.226	0.0008	1.884	Purifying
144	1.595	0.362	0.599	0.947	0.3306	0.941	Neutral
145	2.070	0.230	0.569	3.166	0.0752	0.894	Purifying
146	1.656	0.271	0.733	2.781	0.0954	1.151	Purifying
147	0.000	0.308	0.205	0.782	0.3765	0.322	Neutral
148	3.559	0.771	1.636	3.127	0.0770	2.569	Purifying
149	0.000	0.000	0.000	0.000	1.0000	0.000	Invariable
150	0.000	0.000	0.000	0.000	1.0000	0.000	Invariable
151	0.470	0.000	0.183	1.879	0.1704	0.288	Neutral
152	1.043	0.355	0.545	0.550	0.4584	0.856	Neutral

153	0.000	0.000	0.000	0.000	1.0000	0.000	Invariable
154	0.000	0.000	0.000	0.000	1.0000	0.000	Invariable
155	0.000	0.000	0.000	0.000	1.0000	0.000	Invariable
156	0.000	0.252	0.157	0.929	0.3350	0.246	Neutral
157	4.915	0.233	0.979	8.737	0.0031	1.538	Purifying
158	0.000	0.000	0.000	0.000	1.0000	0.000	Invariable
159	1.192	0.320	0.500	0.744	0.3885	0.785	Neutral
160	1.258	0.472	0.607	0.164	0.6854	0.953	Neutral
161	2.406	0.000	0.550	8.322	0.0039	0.864	Purifying
162	0.000	0.000	0.000	0.000	1.0000	0.000	Invariable
163	0.000	0.308	0.205	0.785	0.3756	0.321	Neutral
164	1.393	0.000	0.292	2.914	0.0878	0.459	Purifying
165	1.913	0.365	0.613	1.178	0.2778	0.963	Neutral
166	1.450	0.373	0.784	1.405	0.2360	1.231	Neutral
167	0.008	0.669	0.524	0.991	0.3195	0.823	Neutral
168	0.633	0.000	0.201	2.239	0.1346	0.316	Neutral
169	2.093	0.000	0.545	4.958	0.0260	0.857	Purifying
170	3.630	0.297	0.740	3.715	0.0539	1.162	Purifying
171	0.000	0.000	0.000	0.000	1.0000	0.000	Invariable
172	0.000	0.000	0.000	0.000	1.0000	0.000	Invariable
173	0.000	0.000	0.000	0.000	1.0000	0.000	Invariable
174	0.722	0.522	0.577	0.061	0.8045	0.906	Neutral
175	1.244	0.000	0.346	5.040	0.0248	0.543	Purifying
176	1.508	0.000	0.346	5.631	0.0176	0.544	Purifying
177	0.000	0.000	0.000	0.000	1.0000	0.000	Invariable
178	0.856	0.000	0.244	2.508	0.1133	0.383	Neutral
179	0.000	0.384	0.262	0.755	0.3850	0.412	Neutral
180	0.000	0.666	0.536	0.868	0.3514	0.842	Neutral
181	0.860	0.000	0.187	3.031	0.0817	0.294	Purifying
182	0.007	0.754	0.532	1.503	0.2201	0.835	Neutral
183	1.941	0.000	0.300	3.622	0.0570	0.471	Purifying

184	0.000	0.810	0.547	2.298	0.1296	0.859	Neutral
185	0.552	0.000	0.221	1.827	0.1765	0.347	Neutral
186	2.440	0.000	0.615	6.484	0.0109	0.966	Purifying
187	0.737	0.243	0.365	0.565	0.4521	0.574	Neutral
188	0.000	0.000	0.000	0.000	1.0000	0.000	Invariable
189	0.000	0.570	0.379	1.622	0.2028	0.595	Neutral
190	0.558	0.000	0.173	2.307	0.1288	0.272	Neutral
191	1.671	0.000	0.442	5.232	0.0222	0.695	Purifying
192	0.846	0.248	0.423	0.836	0.3607	0.664	Neutral
193	1.609	0.000	0.281	3.460	0.0629	0.442	Purifying
194	3.599	0.000	0.795	11.087	0.0009	1.248	Purifying
195	0.000	2.591	2.186	1.792	0.1807	3.433	Neutral
196	1.378	0.000	0.260	3.137	0.0765	0.409	Purifying
197	1.839	0.000	0.604	6.470	0.0110	0.948	Purifying
198	1.283	0.000	0.473	3.798	0.0513	0.743	Purifying
199	0.470	0.000	0.197	1.734	0.1879	0.309	Neutral
200	2.036	0.000	0.451	5.652	0.0174	0.708	Purifying
201	1.542	0.000	0.429	4.971	0.0258	0.674	Purifying
202	3.713	0.355	0.894	3.632	0.0567	1.404	Purifying
203	0.870	0.000	0.359	3.501	0.0613	0.563	Purifying
204	1.827	0.000	0.451	5.439	0.0197	0.709	Purifying
205	0.374	0.000	0.167	1.595	0.2065	0.263	Neutral
206	0.000	0.000	0.000	0.000	1.0000	0.000	Invariable
207	1.410	0.613	0.765	0.372	0.5421	1.201	Neutral
208	1.512	0.000	0.366	5.627	0.0177	0.574	Purifying
209	1.168	0.758	0.924	0.170	0.6804	1.451	Neutral
210	1.479	1.083	1.220	0.108	0.7422	1.916	Neutral
211	0.964	0.749	0.843	0.060	0.8059	1.323	Neutral
212	0.666	0.000	0.206	2.327	0.1272	0.323	Neutral
213	0.855	0.000	0.222	2.680	0.1016	0.348	Neutral
214	5.524	0.000	0.797	13.825	0.0002	1.251	Purifying

215	0.717	0.305	0.430	0.347	0.5556	0.674	Neutral
216	1.797	0.000	0.379	6.105	0.0135	0.595	Purifying
217	0.000	0.000	0.000	0.000	1.0000	0.000	Invariable
218	0.000	0.307	0.178	1.081	0.2984	0.280	Neutral
219	0.000	0.000	0.000	0.000	1.0000	0.000	Invariable
220	0.000	0.344	0.243	0.691	0.4058	0.382	Neutral
221	1.821	0.000	0.379	6.126	0.0133	0.596	Purifying
222	0.432	0.000	0.177	1.777	0.1826	0.278	Neutral
223	0.000	0.000	0.000	0.000	1.0000	0.000	Invariable
224	1.367	0.237	0.448	1.674	0.1958	0.704	Neutral
225	0.000	0.000	0.000	0.000	1.0000	0.000	Invariable
226	2.098	0.000	0.728	5.894	0.0152	1.142	Purifying
227	0.000	0.000	0.000	0.000	1.0000	0.000	Invariable
228	1.542	0.310	0.763	2.104	0.1469	1.198	Neutral
229	3.058	0.364	0.907	2.973	0.0847	1.424	Purifying
230	0.000	0.000	0.000	0.000	1.0000	0.000	Invariable
231	0.737	0.355	0.478	0.258	0.6115	0.750	Neutral
232	1.489	0.000	0.496	4.344	0.0371	0.779	Purifying
233	0.714	0.349	0.470	0.252	0.6156	0.737	Neutral
234	0.718	0.342	0.465	0.252	0.6157	0.731	Neutral
235	1.197	0.000	0.516	4.898	0.0269	0.810	Purifying
236	0.000	0.000	0.000	0.000	1.0000	0.000	Invariable
237	0.000	0.245	0.202	0.380	0.5378	0.318	Neutral
238	0.813	0.000	0.218	2.613	0.1060	0.343	Neutral
239	0.000	0.344	0.243	0.692	0.4055	0.382	Neutral
240	1.705	0.000	0.375	5.959	0.0146	0.589	Purifying
241	4.320	0.237	1.147	9.498	0.0021	1.801	Purifying
242	0.552	0.000	0.221	1.827	0.1765	0.347	Neutral
243	2.679	0.000	0.456	6.737	0.0094	0.715	Purifying
244	1.741	0.358	0.601	1.079	0.2989	0.943	Neutral
245	1.059	0.000	0.194	3.316	0.0686	0.305	Purifying

246	0.813	0.000	0.240	2.429	0.1191	0.377	Neutral
247	0.000	0.000	0.000	0.000	1.0000	0.000	Invariable
248	0.000	0.296	0.235	0.457	0.4992	0.369	Neutral
249	0.000	0.000	0.000	0.000	1.0000	0.000	Invariable
250	0.000	0.000	0.000	0.000	1.0000	0.000	Invariable
251	0.000	0.000	0.000	0.000	1.0000	0.000	Invariable
252	1.043	0.000	0.319	3.241	0.0718	0.501	Purifying
253	0.863	1.225	1.135	0.081	0.7761	1.781	Neutral
254	5.970	0.000	0.953	12.664	0.0004	1.496	Purifying
255	0.000	0.000	0.000	0.000	1.0000	0.000	Invariable
256	1.171	0.000	0.429	3.915	0.0479	0.674	Purifying
257	0.813	0.000	0.219	2.609	0.1062	0.344	Neutral
258	1.390	0.374	0.729	1.183	0.2768	1.144	Neutral
259	0.000	0.297	0.235	0.458	0.4984	0.369	Neutral
260	0.008	0.403	0.394	0.099	0.7533	0.619	Neutral
261	1.525	0.000	0.368	5.644	0.0175	0.577	Purifying
262	1.533	0.000	0.363	5.582	0.0182	0.571	Purifying
263	0.000	0.000	0.000	0.000	1.0000	0.000	Invariable
264	2.235	0.000	0.377	6.675	0.0098	0.592	Purifying
265	1.338	0.000	0.260	3.103	0.0782	0.408	Purifying
266	0.000	0.000	0.000	0.000	1.0000	0.000	Invariable
267	0.000	0.261	0.187	0.665	0.4147	0.293	Neutral
268	0.875	0.725	0.767	0.022	0.8811	1.205	Neutral
269	1.395	0.000	0.459	4.375	0.0365	0.720	Purifying
270	0.666	0.000	0.222	2.135	0.1440	0.349	Neutral
271	1.944	0.230	0.558	3.040	0.0812	0.875	Purifying
272	1.457	0.307	0.747	2.048	0.1524	1.174	Neutral
273	0.000	0.000	0.000	0.000	1.0000	0.000	Invariable
274	1.177	0.300	0.598	1.292	0.2556	0.940	Neutral
275	0.633	0.000	0.201	2.248	0.1338	0.316	Neutral
276	0.966	0.342	0.506	0.491	0.4836	0.794	Neutral

277	0.713	0.000	0.231	2.259	0.1328	0.362	Neutral
278	4.696	0.504	1.248	6.221	0.0126	1.960	Purifying
279	1.402	0.000	0.548	5.446	0.0196	0.860	Purifying
280	1.998	0.374	0.866	1.963	0.1612	1.359	Neutral
281	0.000	0.000	0.000	0.000	1.0000	0.000	Invariable
282	1.389	0.334	0.531	0.843	0.3586	0.834	Neutral
283	0.010	0.398	0.393	0.057	0.8106	0.617	Neutral
284	1.499	0.000	0.471	4.569	0.0326	0.740	Purifying
285	1.232	0.000	0.224	3.306	0.0690	0.352	Purifying
286	3.446	0.342	0.878	3.479	0.0622	1.379	Purifying
287	0.000	0.000	0.000	0.000	1.0000	0.000	Invariable
288	0.000	0.507	0.387	1.056	0.3041	0.608	Neutral
289	0.000	0.344	0.219	0.905	0.3413	0.344	Neutral
290	0.000	0.000	0.000	0.000	1.0000	0.000	Invariable
291	1.822	0.000	0.602	6.463	0.0110	0.946	Purifying
292	1.462	0.000	0.363	5.453	0.0195	0.570	Purifying
293	0.000	0.316	0.207	0.843	0.3586	0.325	Neutral
294	1.050	0.000	0.270	2.799	0.0943	0.424	Purifying
295	3.649	0.000	0.535	6.747	0.0094	0.840	Purifying
296	2.539	0.238	0.739	4.621	0.0316	1.160	Purifying
297	1.411	0.000	0.481	4.182	0.0409	0.755	Purifying
298	0.000	1.117	0.699	2.809	0.0937	1.098	Diversifying
299	2.787	0.304	0.898	3.925	0.0476	1.410	Purifying
300	0.000	0.000	0.000	0.000	1.0000	0.000	Invariable
301	1.706	0.788	1.050	0.727	0.3938	1.649	Neutral
302	1.043	0.000	0.342	4.372	0.0365	0.537	Purifying
303	2.771	0.733	0.978	0.953	0.3289	1.536	Neutral
304	2.170	0.000	0.736	5.998	0.0143	1.155	Purifying
305	0.000	0.694	0.486	1.371	0.2416	0.763	Neutral
306	0.553	0.719	0.653	0.042	0.8383	1.026	Neutral
307	10.187	0.000	0.879	16.463	0.0000	1.380	Purifying

308	0.008	0.695	0.563	0.830	0.3623		0.884	Neutral
309	0.007	0.804	0.661	0.745	0.3882		1.038	Neutral
310	0.556	0.367	0.442	0.084	0.7722		0.694	Neutral
311	1.619	0.000	0.286	3.423	0.0643		0.449	Purifying
312	1.342	0.000	0.422	4.406	0.0358		0.663	Purifying
313	1.509	0.000	0.473	4.609	0.0318		0.743	Purifying
314	1.492	0.000	0.430	4.928	0.0264		0.676	Purifying
315	1.448	0.250	0.551	2.079	0.1493		0.866	Neutral
316	0.000	0.000	0.000	0.000	1.0000		0.000	Invariable
317	52.214	0.189	0.190	0.064	0.8005		0.298	Neutral
318	2.399	0.000	0.801	8.420	0.0037		1.258	Purifying
319	0.000	0.297	0.235	0.458	0.4984		0.369	Neutral
320	0.000	0.000	0.000	0.000	1.0000		0.000	Invariable
321	0.552	0.000	0.194	2.088	0.1485		0.305	Neutral
322	2.147	0.000	0.769	7.887	0.0050		1.207	Purifying
323	1.499	0.000	0.508	6.162	0.0131		0.798	Purifying
324	2.273	0.000	0.632	7.520	0.0061		0.992	Purifying
325	0.000	0.366	0.302	0.381	0.5370		0.474	Neutral
326	0.470	0.000	0.197	1.734	0.1879		0.309	Neutral
327	1.748	0.337	0.565	1.136	0.2864		0.887	Neutral
328	0.012	0.252	0.209	0.340	0.5599		0.329	Neutral
329	0.000	1.356	1.227	0.546	0.4600		1.927	Neutral
330	1.543	0.301	0.674	1.875	0.1709		1.059	Neutral
331	1.741	0.000	0.295	3.518	0.0607		0.463	Purifying
332	0.000	1.131	0.717	2.706	0.1000		1.126	Diversifying
333	3.576	0.618	1.313	3.706	0.0542		2.062	Purifying
334	0.468	0.309	0.372	0.090	0.7646		0.584	Neutral
335	0.000	0.000	0.000	0.000	1.0000		0.000	Invariable
336	0.394	0.000	0.171	1.626	0.2022		0.269	Neutral
337	0.000	0.712	0.586	0.774	0.3791		0.920	Neutral
338	1.378	0.000	0.208	3.811	0.0509		0.327	Purifying

339	0.970	0.000	0.374	3.787	0.0516	0.587	Purifying
340	1.181	0.229	0.382	1.144	0.2848	0.600	Neutral
341	0.713	0.000	0.230	2.266	0.1322	0.361	Neutral
342	0.713	0.000	0.211	2.436	0.1186	0.331	Neutral
343	0.643	0.339	0.445	0.188	0.6644	0.699	Neutral
344	6.512	0.000	1.990	17.758	0.0000	3.124	Purifying
345	0.875	0.000	0.250	2.572	0.1088	0.392	Neutral
346	1.379	0.000	0.211	3.558	0.0592	0.331	Purifying
347	0.000	0.000	0.000	0.000	1.0000	0.000	Invariable
348	2.320	0.356	0.634	1.536	0.2152	0.996	Neutral
349	1.997	0.225	0.559	3.109	0.0779	0.878	Purifying
350	0.652	0.280	0.389	0.340	0.5598	0.610	Neutral
351	0.813	0.000	0.219	2.609	0.1062	0.344	Neutral
352	0.000	0.000	0.000	0.000	1.0000	0.000	Invariable
353	1.292	0.305	0.625	1.441	0.2300	0.981	Neutral
354	0.689	0.000	0.210	2.346	0.1256	0.330	Neutral
355	0.970	0.000	0.373	3.794	0.0514	0.585	Purifying
356	0.000	0.000	0.000	0.000	1.0000	0.000	Invariable
357	2.275	0.362	0.617	1.419	0.2335	0.969	Neutral
358	0.000	0.304	0.222	0.621	0.4307	0.349	Neutral
359	0.000	0.236	0.196	0.381	0.5369	0.308	Neutral
360	0.689	0.000	0.210	2.346	0.1256	0.330	Neutral
361	0.000	0.350	0.245	0.697	0.4039	0.384	Neutral
362	0.560	0.000	0.210	1.936	0.1641	0.330	Neutral
363	1.280	0.397	0.602	0.621	0.4307	0.945	Neutral
364	0.000	0.000	0.000	0.000	1.0000	0.000	Invariable
365	7.390	0.246	1.067	10.928	0.0009	1.675	Purifying
366	2.673	1.123	1.566	1.201	0.2731	2.459	Neutral
367	0.724	0.268	0.392	0.455	0.5002	0.616	Neutral
368	3.853	0.000	0.575	7.247	0.0071	0.902	Purifying
369	0.000	0.000	0.000	0.000	1.0000	0.000	Invariable

370	1.247	0.000	0.196	3.568	0.0589	0.308	Purifying
371	0.000	0.235	0.205	0.289	0.5908	0.322	Neutral
372	0.633	0.000	0.201	2.239	0.1345	0.316	Neutral
373	4.083	0.304	1.035	5.961	0.0146	1.625	Purifying
374	0.000	0.000	0.000	0.000	1.0000	0.000	Invariable
375	0.712	0.520	0.571	0.066	0.7967	0.897	Neutral
376	0.718	0.341	0.465	0.263	0.6078	0.729	Neutral
377	0.000	0.000	0.000	0.000	1.0000	0.000	Invariable
378	1.382	0.000	0.240	4.342	0.0372	0.377	Purifying
379	0.000	0.303	0.202	0.809	0.3684	0.316	Neutral
380	1.525	0.000	0.423	4.889	0.0270	0.665	Purifying
381	0.557	0.219	0.317	0.402	0.5261	0.497	Neutral
382	0.740	0.375	0.496	0.220	0.6389	0.779	Neutral
383	0.000	0.303	0.201	0.808	0.3688	0.316	Neutral
384	3.853	0.000	0.563	7.322	0.0068	0.883	Purifying
385	2.088	0.000	0.504	5.415	0.0200	0.791	Purifying
