# Supplementary material for: Molecular Evolution of the Chikungunya Virus E1 Gene in Saudi Arabia: Predominance of Purifying Selection and ECSA/IOL Lineage Circulation
Source: Viruses. 2026 Jul 19;18(7):791. doi: 10.3390/v18070791 (PMC13431504; doi:10.3390/v18070791)
Supplement: Supplementary file 1 [file viruses-18-00791-s001.zip › Section S3.4 (FUBAR, FEL, MEME, SLAC).zip!/FUBAR.zip!/FUBAR-Plot.pdf]

Fast Unconstrained Bayesian AppRoximation

results summary

INPUT DATA | **user.nex** | **109** sequences | **385** sites

FUBAR **found evidence** of

- pervasive positive/diversifying selection at 1 sites
- pervasive negative/purifying selection at 140 sites

with posterior probability of 0.9 .

See here for more information about the FUBAR method.

Please cite PMID 23420840 if you use this result in a publication, presentation, or other scientific work.
