# Supplementary material for: Molecular Evolution of the Chikungunya Virus E1 Gene in Saudi Arabia: Predominance of Purifying Selection and ECSA/IOL Lineage Circulation
Source: Viruses. 2026 Jul 19;18(7):791. doi: 10.3390/v18070791 (PMC13431504; doi:10.3390/v18070791)
Supplement: Supplementary file 1 [file viruses-18-00791-s001.zip › Section S3.4 -Figures S5.zip!/Figure S5.pdf]

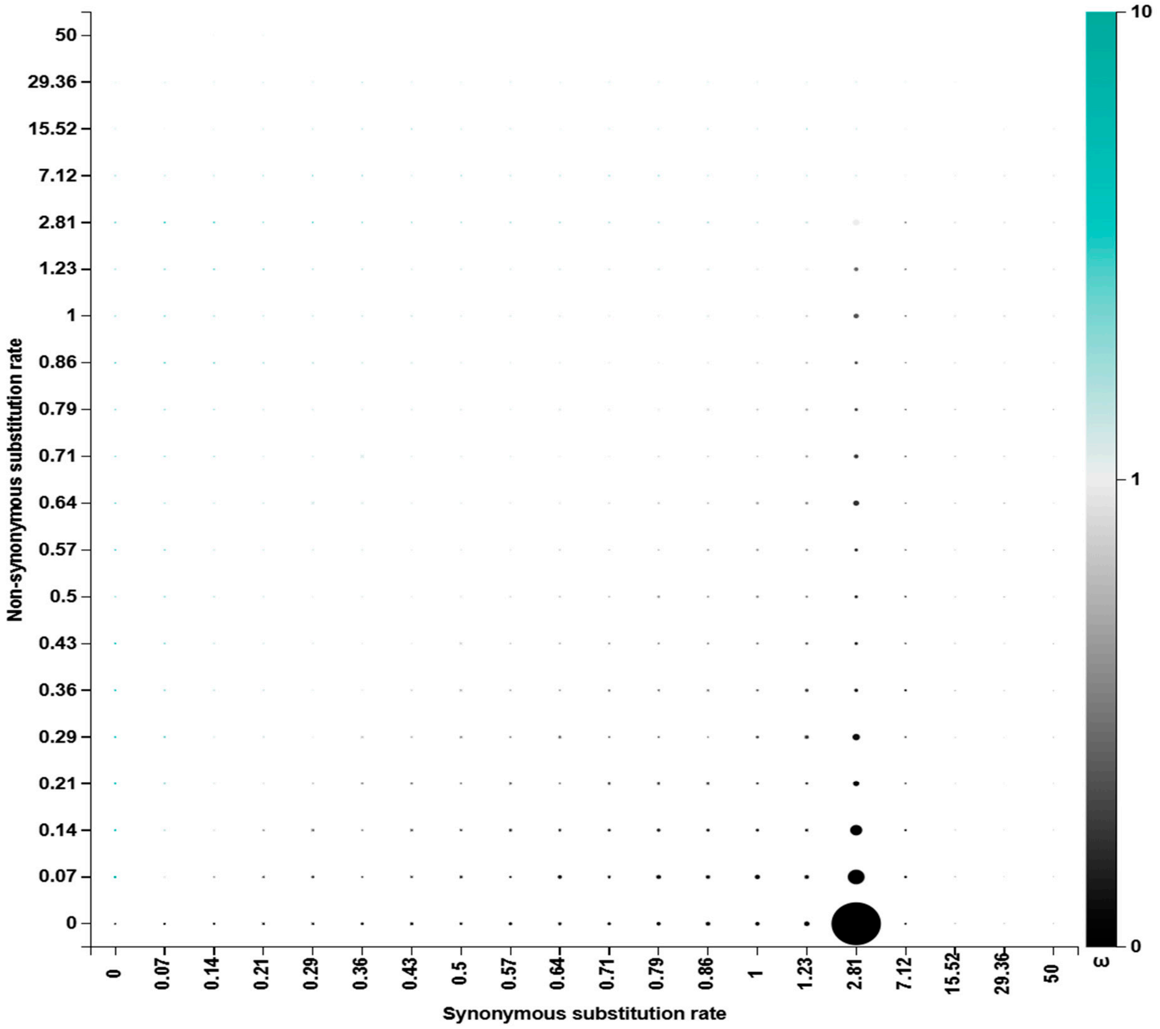

Figure S5. FUBAR analysis of selection pressures acting on the CHIKV *E1* glycoprotein. The figure displays the posterior distribution over the discretized rate grid for the 385 codons of the *E1* gene, inferred using the Fast Unconstrained Bayesian AppRoximation (FUBAR) method implemented in the Datamonkey 2.0 web server. Each cell in the grid represents the posterior probability that a given codon (y-axis, shown as codon positions 1–385) falls into a specific rate category of non-synonymous substitution (*dN*, x-axis). Dot size is proportional to the posterior weight allocated to each gridpoint, and color intensity indicates the strength of selection: darker shades (blue) indicate stronger purifying selection ($dN/dS < 1$), while lighter shades indicate neutrality ($dN/dS \approx 1$). No codon was identified under pervasive positive selection at the predefined threshold of posterior probability ≥ 0.9. FUBAR identified 140 sites under purifying selection (PP ≥ 0.9), with the strongest signals at codons 135, 307, and 344 (PP = 1.000). The highest posterior

probability for positive selection was observed at codon 195 (PP = 0.022), which is well below the cutoff. The analysis used default settings (MCMC chains = 2, burn-in = 500, sampling = 2500, substitution model = MG94).
