# Supplementary material for: Molecular Evolution of the Chikungunya Virus E1 Gene in Saudi Arabia: Predominance of Purifying Selection and ECSA/IOL Lineage Circulation
Source: Viruses. 2026 Jul 19;18(7):791. doi: 10.3390/v18070791 (PMC13431504; doi:10.3390/v18070791)
Supplement: Supplementary file 1 [file viruses-18-00791-s001.zip › Section S3.5 -Figures S1-S4.zip!/Figure S6.pdf]

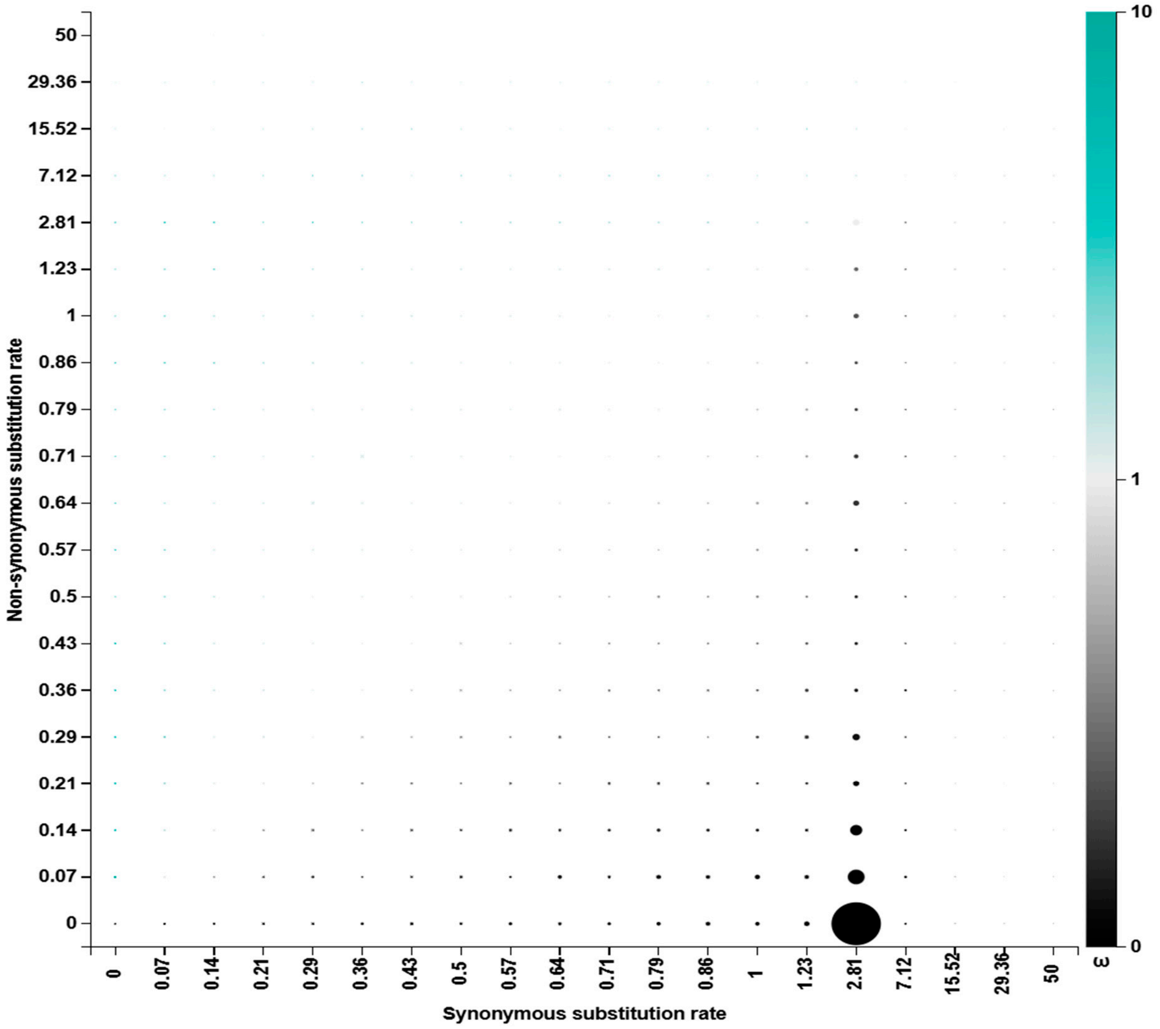

Figure S6. FUBAR analysis of selection pressures acting on the CHIKV E1 glycoprotein. The graph shows the posterior distribution over the discretized rate grid, where dot size is proportional to the posterior weight allocated to each gridpoint, and color indicates the intensity of selection. Darker blue = stronger purifying selection; lighter shades = neutrality. FUBAR identified 140 sites under purifying selection (PP ≥ 0.9) and codon 195 under pervasive positive selection (PP = 0.942, Bayes Factor = 41.307).
