# Supplementary material for: Molecular Evolution of the Chikungunya Virus E1 Gene in Saudi Arabia: Predominance of Purifying Selection and ECSA/IOL Lineage Circulation
Source: Viruses. 2026 Jul 19;18(7):791. doi: 10.3390/v18070791 (PMC13431504; doi:10.3390/v18070791)
Supplement: Supplementary file 1 [file viruses-18-00791-s001.zip › Section S3.4 (FUBAR, FEL, MEME, SLAC).zip!/SLAC.zip!/SLAC.pdf]

INPUT DATA | user.nex | 109 sequences | 385 sites

Export

SLAC **found evidence** of pervasive

positive/diversifying selection at 0 sites

negative/purifying selection at 58 sites

with p-value threshold of 0.1.

See here for more information about the SLAC method.

Please cite PMID 15703242 if you use this result in a publication, presentation, or other scientific work.

Partition information

Partition	Sites	Branches		Branch Length			Selected at p≤0.1	
		Tested	Total	Tested	% of total	Total	Positive	Negative
1	385	155	155	0.000913	0.136%	0.669	0	58

Model fits

Model	AIC_C	log L	Parameters	Rate distributions
Nucleotide GTR	11640.09	-5596.65	223	
Global MG94xREV	11008.85	-5333.73	170	non-synonymous/synonymous rate ratio for *test*: 100% @ 0.208

Execution time

Task	Time	%
Total time	00:02:16	
Model fitting	00:00:04	2.94%
Primary SLAC analysis	00:00:02	1.47%
Ancestor sampling analysis	00:02:10	95.59%
